# CXCL10 deficiency limits macrophage infiltration, preserves lung matrix, and enables lung growth in bronchopulmonary dysplasia

**DOI:** 10.1186/s41232-023-00301-6

**Published:** 2023-10-24

**Authors:** Dharmesh V. Hirani, Florian Thielen, Siavash Mansouri, Soula Danopoulos, Christina Vohlen, Pinar Haznedar-Karakaya, Jasmine Mohr, Rebecca Wilke, Jaco Selle, Thomas Grosch, Ivana Mizik, Margarete Odenthal, Cristina M. Alvira, Celien Kuiper-Makris, Gloria S. Pryhuber, Christian Pallasch, S. van Koningsbruggen-Rietschel, Denise Al-Alam, Werner Seeger, Rajkumar Savai, Jörg Dötsch, Miguel A. Alejandre Alcazar

**Affiliations:** 1 Department of Pediatric and Adolescent Medicine, Translational Experimental Pediatrics, Experimental Pulmonology, University Hospital Cologne, Faculty of Medicine, University of Cologne, Kerpener Strasse 62, Cologne, 50937 Germany; 2 Universities of Giessen and Marburg Lung Center (UGMLC), member of the German Center for Lung Research (DZL), Institute for Lung Health (ILH) and Cardio-Pulmonary Institute (CPI), Gießen, Germany; 3 Department of Lung Development and Remodeling, Max-Planck-Institute for Heart and Lung Research, Member of the German Center for Lung Research (DZL), Bad Nauheim, Germany; 4 Lundquist Institute for Biomedical Innovation at Harbor-UCLA Medical Center, Torrance, CA USA; 5 Department of Pediatric and Adolescent Medicine, Faculty of Medicine, University Hospital Cologne, and University of Cologne, Cologne, Germany; 6 Center for Molecular Medicine Cologne (CMMC), University Hospital Cologne, Faculty of Medicine, and University of Cologne, Cologne, Germany; 7 Institute for Pathology, University Hospital Cologne, Faculty of Medicine, and University of Cologne, Cologne, Germany; 8 Department of Pediatrics, Stanford University School of Medicine, Stanford, CA USA; 9 Department of Pediatrics, Division of Neonatology, University of Rochester Medical Center, Rochester, NY USA; 10 Department I of Internal Medicine, Center for Integrated Oncology (CIO) Köln-Bonn, University of Cologne, Cologne, Germany; 11 Cologne Excellence Cluster On Stress Responses in Aging-Associated Diseases (CECAD), University Hospital of Cologne, University of Cologne, Cologne, Germany

**Keywords:** CXCL10, Hyperoxia, Elastic fibers, Collagen, Lung matrix remodeling, Bronchopulmonary dysplasia

## Abstract

Preterm infants with oxygen supplementation are at high risk for bronchopulmonary dysplasia (BPD), a neonatal chronic lung disease. Inflammation with macrophage activation is central to the pathogenesis of BPD. CXCL10, a chemotactic and pro-inflammatory chemokine, is elevated in the lungs of infants evolving BPD and in hyperoxia-based BPD in mice. Here, we tested if CXCL10 deficiency preserves lung growth after neonatal hyperoxia by preventing macrophage activation. To this end, we exposed *Cxcl10* knockout (*Cxcl10*^−/−^) and wild-type mice to an experimental model of hyperoxia (85% O_2_)-induced neonatal lung injury and subsequent regeneration. In addition, cultured primary human macrophages and murine macrophages (J744A.1) were treated with CXCL10 and/or CXCR3 antagonist. Our transcriptomic analysis identified CXCL10 as a central hub in the inflammatory network of neonatal mouse lungs after hyperoxia. Quantitative histomorphometric analysis revealed that *Cxcl10*^−/−^ mice are in part protected from reduced alveolar. These findings were related to the preserved spatial distribution of elastic fibers, reduced collagen deposition, and protection from macrophage recruitment/infiltration to the lungs in *Cxcl10*^−/−^ mice during acute injury and regeneration. Complimentary, studies with cultured human and murine macrophages showed that hyperoxia induces *Cxcl10* expression that in turn triggers M1-like activation and migration of macrophages through CXCR3. Finally, we demonstrated a temporal increase of macrophage-related CXCL10 in the lungs of infants with BPD. In conclusion, our data demonstrate macrophage-derived CXCL10 in experimental and clinical BPD that drives macrophage chemotaxis through CXCR3, causing pro-fibrotic lung remodeling and arrest of alveolarization. Thus, targeting the CXCL10-CXCR3 axis could offer a new therapeutic avenue for BPD.

**Supplementary Information:**

The online version contains supplementary material available at 10.1186/s41232-023-00301-6.

## Introduction

Bronchopulmonary dysplasia (BPD) is a chronic lung disease of the premature infant who often requires life-saving respiratory support such as supplemental oxygen and/or mechanical ventilation due to the immaturity of the lung. Despite significant advances in neonatal management and improved survival, BPD still occurs in up to 40% of infants born less than 29 weeks gestation [1]. Lungs of infants with BPD are characterized by alveolar and vascular hypoplasia that results in an emphysema-like lung structure that is evident beyond infancy. Extracellular matrix (ECM) is essential for alveolar formation by providing the "framework" for resident lung cells, regulating cellular signaling, and maintaining lung elasticity as well as the alveolar integrity [2, 3]. In contrast, perturbed assembly and increased degradation of the lung matrix favoring fibrosis are hallmarks of severe BPD [4–7].

Inflammation is central in the pathogenesis of BPD and a main regulator of lung matrix remodeling, but the mechanisms remain elusive. Previous studies have shown elevated concentrations of pro-inflammatory cytokines and chemokines, e.g., IL17, CCl7, CXCL5, and CCL2, in the lungs of infants with BPD [8–10]. Recent studies from our group demonstrated an activation of macrophages along with IL-6 signaling in human lungs with BPD as well as in the lungs of neonatal mice exposed to prolonged hyperoxia as a model of the BPD [8]. Excessive activation and recruitment of macrophages in murine lungs after hyperoxia caused apoptosis of epithelial cells, reduced alveolarization, and failed angiogenesis, ultimately aggravating the pathogenesis of BPD and resulting in a poor clinical outcome [10, 11]. Moreover, cross-talk of activated macrophages with fibroblasts also accompanies perturbed and increased ECM deposition [12–18] and might lead to irreversible matrix changes with aberrant lung growth in neonatal mice.

Various factors trigger the migration of activated macrophages in the lung, e.g., chemokines and cell adhesion molecules [19]. For example, the C-X-C motif chemokine 10 (CXCL10), also known as IFN-γ–induced protein (IP-10), binds to the CXCR3 receptor, triggers chemotaxis, cell growth as well as apoptosis [20, 21]. CXCL10 also modulates immune responses by recruiting inflammatory cells [i.e., neutrophils, T lymphocytes, and natural killer (NK) cells] to the sites of inflammation [22, 23]. Interestingly, CXCL10 is not only secreted by macrophages but also activates and attracts macrophages into tissues, and thus CXCL10 substantially controls the inflammatory response as well as the tissue and matrix homeostasis [24–26]. In contrast, either neutralization of CXCL10 or blockage of CXCR3 reduces the recruitment of immune cells, e.g., macrophages, thereby mitigating the inflammatory pulmonary and perivascular response [19, 27]. Initial studies indicate a controversial role of CXCL10 in the fibrotic processes. While CXCL10 has been described to act as an anti-fibrotic chemokine in an experimental model of bleomycin-induced lung fibrosis, it promotes liver fibrosis [28, 29]. However, the functional role of CXCL10 in BPD has not been addressed to date and remains elusive.

In the present study, the transcriptomic analysis identified an inflammatory profile in neonatal lungs after hyperoxia with a marked lung-intrinsic increase of *Cxcl10* together with macrophage recruitment to the lung. In contrast, transgenic mice with *Cxcl10* deficiency were in part protected from macrophage influx to the lung, epithelial cell apoptosis, loss of alveolar epithelial type 2 cells (AT2), perturbed elastic fiber deposition, and increased collagen deposition. Of note, these findings were not only found in hyperoxia-induced acute injury at postnatal day 14 (P14) but persisted during regeneration in normoxia at P28. Mechanistically, we show that the blockage of the CXCR3 receptor reduces the CXCL10-mediated human and murine macrophage invasion. Finally, the lungs of infants with BPD showed an age-dependent increase of macrophages that was related to a macrophage-specific elevation of CXCL10.

## Results

### Transcriptomic analysis identified *Cxcl10* as a central hub in hyperoxia-induced inflammation in newborn mice at postnatal day 14 (P14)

**Fig. 1:**
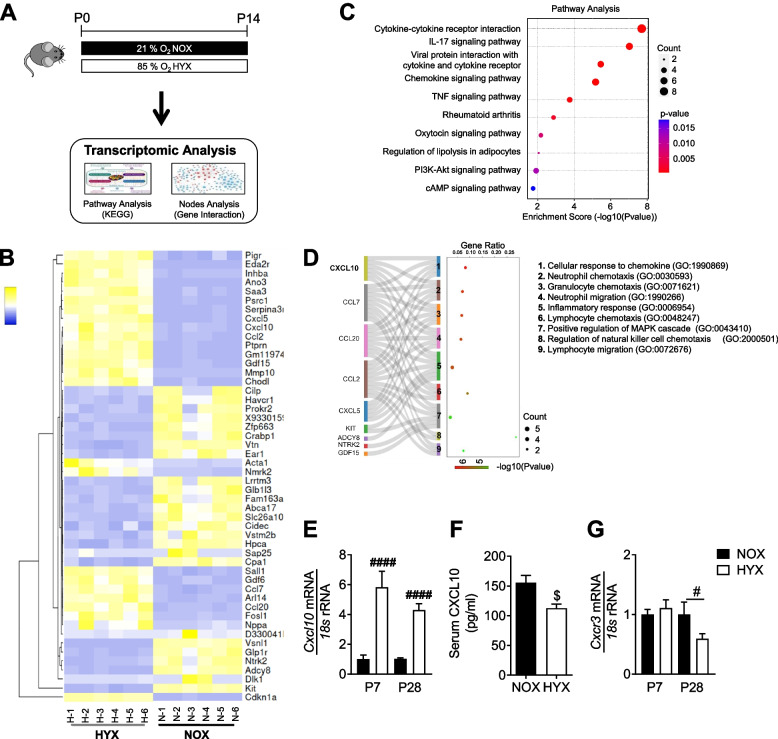
CXCL10 is a central hub of the inflammatory network of hyperoxia-induced lung injury in newborn mice. **A** Experimental setup for transcriptomic analysis of lungs of newborn mice exposed to normoxia (21% O_2_, NOX) or hyperoxia (80% O_2_, HYX) from birth until postnatal day 14 (P14). **B**, **C**: Heatmap (**B**) and pathways analysis (**C**). The heatmap contains the top 25 up- and downregulated differentially expressed genes (DEG; *p* < 0.05 and log fold change > 1.5), *n* = 6 per group. **D** Sneaky plot for DEGs association with (GO) Biological Process. **E** Assessment of gene expression of *Cxcl10* (C-X-C motif chemokine 10) using quantitative RT-PCR in lungs of newborn mice exposed to NOX (black bars) or HYX (white bars) from birth until P7 or P28; *n* = 11–12 per group. **F** Quantification of CXCL10 protein concentration using ELISA in serum of mice exposed to NOX or HYX from birth until P14. **G** Measurement of mRNA expression of *Cxcr3* (C-X-C Motif Chemokine Receptor 3) in lungs exposed to NOX or HYX from birth until P7 or 28. Mean ± SEM; *n* = 3–6/group; Mann–Whitney test: #*p* < 0.05; ####*p* < 0.0001, Unpaired t test $*p* < 0.05

Hyperoxia promotes inflammation through the alteration of cytokine and chemokine signaling pathways in the lungs of neonatal mice. We recently identified an important role of macrophages in the pathogenesis of BPD using the RNA sequencing (RNA-Seq)-based transcriptomic profiling [8]. To further investigate central signaling hubs in the lung inflammatory response after hyperoxia, we now analyzed our publicly available lung bulk RNA-Seq data from newborn mice exposed to hyperoxia for 14 days (Fig. 1A). In the present study, the top 25 up and down-regulated differentially expressed genes (DEG) (Supplementary Table 1) were taken into account as depicted in the heatmap in Fig. 1B. Subsequently, an online open-source platform called SRPLOT (https://www.bioinformatics.com.cn/en) was used for in-silico data analysis and visualization. We performed KEGG pathway annotation analysis based on the enrichment score using SRPLOT and found that pathways associated with inflammation, e.g., cytokine-cytokine receptor interaction, chemokine signaling pathways, or IL-17 signaling pathways, are amongst the significantly regulated pathways in lungs of newborn mice after hyperoxia (Fig. 1C). To identify central molecular hubs in the inflammatory network, we used DEGs associated with the top-ten KEGG pathways as input for gene ontology (GO) analysis. We visualized the interaction of the DEGs with the pathways based on GO annotation using a sneaky plot and found central DEGs, notable amongst those *Cxcl10* (Fig. 1D). For validation of these findings, we next assessed gene expression of *Cxcl10* in lungs at postnatal day 7 (P7) and P28 (Fig. 1E) and found a 4- to 6-fold upregulation in neonatal mouse lungs after hyperoxia at both time points. In addition, we performed a serum ELISA for CXCL10 and detected a significant reduction of CXCL10 protein after hyperoxia at P28 (Fig. 1F), indicating a lung-intrinsic upregulation of *Cxcl10* as an inflammation and macrophage-regulating chemokine [19, 22, 23, 27]. In contrast, the expression of *Cxcr3*, the CXCL10 receptor, was not altered between normoxia and hyperoxia at P7, but significantly downregulated at P28 after a prolonged exposure to hyperoxia (Fig. 1G). Collectively, our data indicate that CXCL10 could play a central role in the inflammatory response in neonatal lungs after hyperoxia.

### Loss of CXCL10 enables alveolar formation, reduces apoptosis, and protects alveolar epithelial type 2 cells (AT2) in newborn mice after hyperoxia

**Fig. 2:**
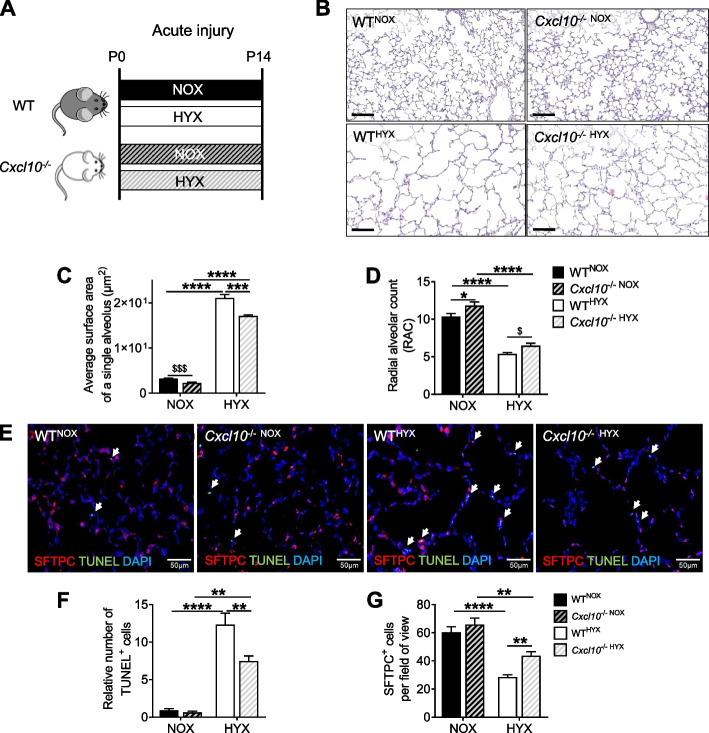
Loss of CXCL10 enables lung growth and preserves survival of alveolar epithelial type 2 cells (AT2) in neonatal mice after hyperoxia. **A** Experimental setup for assessment of lung morphometry and lung cell survival in newborn mice exposed to normoxia (21% O_2_, NOX) or hyperoxia (85% O_2_, HYX) from birth until postnatal day 14 (P14). **B** Representative images of hematoxylin and eosin (H&E) stained lung tissue sections of wildtype (WT) and *Cxcl10* knockout mice (*Cxcl10*^−/−^) at P14. **C**, **D** Quantitative histomorphometric analyses for the average surface area of a single alveolus (**C**) and radial alveolar count (RAC; **D**). **E** Representative co-immunofluorescent staining for surfactant protein **C** (SFTPC; a marker of AT2, red) and TUNEL as an indicator of apoptosis (green) in lungs at P14; DAPI was used for nuclear staining. Quantification of the percentage of TUNEL^+^ cells (**F**) and SFTPC ^+^ cells relative to all DAPI^+^ cells (**G**). Mean ± SEM; *n* = 3–6/group; Unpaired t test: $*p* < 0.05; Two-way ANOVA: *p < 0.05; **p < 0.01; ***p < 0.001; ****p < 0.0001; scale bars: 20X, 40X

Next, we tested if Cxcl10 is mechanistically important in alveolarization and cell survival during acute hyperoxia-induced neonatal lung injury. To this end, wild-type (WT) or *Cxcl10* knockout (*Cxcl10*^−/−^) mice were exposed to normoxia (NOX; 21% O_2_) or hyperoxia (HYX; 85% O_2_) after birth for 14 days, and lungs were studied at P14 as illustrated in Fig. 2A. Neonatal hyperoxia markedly blocked alveolar formation in WT mice as indicated by an increased average surface area of a single alveolus and a reduced radial alveolar count (RAC); in contrast, *Cxcl10*^−/−HYX^ were partially protected from these effects when compared to WT^HYX^ (Fig. 2B-D). In addition, co-immunofluorescent staining for TUNEL (a marker for apoptotic cells) and surfactant protein C (SFTPC; a marker for AT2) showed an almost 10-fold increased number of apoptotic cells in lungs of WT^HYX^ that was significantly attenuated in lungs of *Cxcl10*^−/−HYX^ (Fig. 2E, F). Similarly, the loss of Cxcl10 partially protected from the reduction of SFTPC^+^ cells after hyperoxia by 50% (Fig. 2G). These data indicate a pro-apoptotic effect of CXCL10 in neonatal lungs exposed to prolonged hyperoxia that could in part explain an arrest of alveolarization observed in lungs with BPD.

### Loss of CXCL10 attenuates fibrotic remodeling in hyperoxia-exposed neonatal lungs through preserved spatial elastic fiber distribution and reduced collagen deposition

**Fig. 3:**
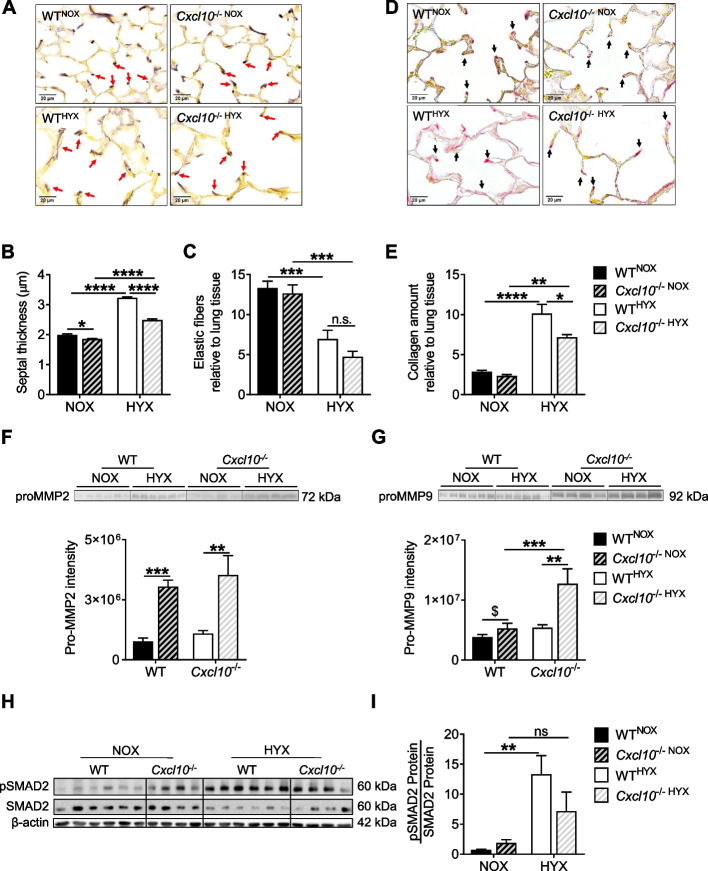
Loss of CXCL10 attenuates fibrotic lung remodeling of neonatal mice after hyperoxia. **A**-**C** Representative lung tissue images showing the spatial distribution of elastic fibers in mouse lungs after exposure to normoxia (21% O_2_, NOX) or hyperoxia (85% O_2_, HYX) from birth until postnatal day 14 (P14) (**A**). Quantitative histomorphometric analysis of septal thickness (**B**). Quantitative elastic fiber content relative to lung tissue (elastic fiber fraction) (**C**). **D**, **E** Representative images of Picro-Sirius red-stained lung sections showing collagen deposition in mouse lungs after exposure to NOX or HYX from birth until P14 (**D**). Quantitative analysis of the collagen amount relative to lung tissue in percentage (%, collagen fraction; **E**). **F**, **G** Gelatin zymography to detect matrix metalloproteinase 2 (MMP2; **F**) and MMP9 (**G**) activity along with densitometric analysis in lungs at P14 after exposure to NOX or HYX. **H**, **I** Immunoblots and respective quantification of phospho-SMAD2 protein (pSMAD2) total SMAD2 protein (downstream effector of TGFβ signaling) in lungs at P14 after exposure to NOX or HYX; β-actin was used as loading control (**H**). Densitometric data summary of pSMAD2 relative to total SMAD2 (**I**). Mean ± SEM; *n* = 4–9/group. Unpaired t test: $*p* < 0.05; Two-way ANOVA: *p < 0.05; **p < 0.01; ***p < 0.001; ****p < 0.0001; scale bar: 100X

Extracellular matrix (ECM) plays an important role in normal lung development and alveolar formation [2, 30]. In contrast, perturbed assembly and distribution of elastic fibers as well as increased collagen production are intimately linked to aberrant alveolarization [6, 7]. Here, we show a significant increase of septal thickness in WT^HYX^, indicating increased ECM deposition. In contrast, *Cxcl10*^−/−NOX^ and *Cxcl10*^−/−HYX^ exhibited a reduced septal thickness when compared to WT^NOX^ and WT^HYX^, respectively (Fig. 3A, B). We next studied the deposition of matrix components in the neonatal lung after hyperoxia. First, we found a reduction of elastic fiber fraction in the lungs of WT^HYX^ and *Cxcl10*^−/−HYX^ when compared to WT^NOX^ and *Cxcl10*^−/−NOX^, respectively. While elastic fibers are predominantly localized at the tip of the secondary crests in lung lungs of WT^NOX^, in neonatal WT^HYX^ lungs, we found them as “brushed-like” structures in the primary septae and not at the tip of the secondary septae as previously described [5]. Interestingly, *Cxcl10*^−/−HYX^ were not protected from the reduced abundance of elastic fibers, but the spatial distribution was predominantly at the tip of the secondary crests (Fig. 3A, C). Since elastic fibers and collagen are interrelated in lung matrix biology, we next performed Sirius red staining as an indicator of collagen. Hyperoxia induced a more than 3-fold increase of collagen in lungs from WT mice, whereas *Cxcl10*^−/−HYX^ was partially protected when compared to WT^HYX^ (Fig. 3D, E). To further understand the mechanisms by which elastic fiber and collagen synthesis as well as deposition are regulated, we assessed the activity of two key matrix metalloproteinases (MMP), MMP2 and MMP9, using zymography. The analysis revealed that hyperoxia significantly induced the activity of MMP2 in both WT and *Cxcl10*^−/−^ mice. In contrast, MMP9 activity was not increased in neonatal lungs of WT^HYX^ when compared to WT^NOX^. Lungs of *Cxcl10*^−/−HYX^, however, exhibited a 2-fold higher MMP9 activity than lungs of *Cxcl10*^−/−NOX^ or WT^NOX^ (Fig. 3F, G). TGFβ signaling plays a central role in lung fibrotic processes and matrix remodeling. Since prior studies from our group showed an activation of the TGFβ pathways in neonatal lungs after hyperoxia, we now assessed phosphorylated SMAD2 (pSMAD2) as a downstream effector of TGFβ [4, 30]. Immunoblot for pSMAD2 and total SMAD2 showed an almost 10-fold activation of pSMAD2 in the lungs of WT^HYX^, whereas this effect was attenuated by almost 50% in lungs of *Cxcl10*^−/−HYX^, indicating a pro-fibrotic function of CXCL10 in neonatal lungs after hyperoxia (Fig. 3H, I). Interestingly, the gene expression of key collagens (*Col1a1*, *Col3a1*, and *Col4a4*) (Supplementary Fig. 1A-C), were not significantly regulated by CXCL10, suggesting that the pro-fibrotic effect might be primarily mediated through an imbalance of lung matrix assembly, deposition, and degradation through proteolytic activity. Collectively, these data show that CXCL10 has a modulatory function on elastic fiber assembly as well as localization and a pro-fibrotic effect through the regulation of MMP9 and TGFβ signaling.

### Loss of CXCL10 limits macrophage influx into neonatal lungs after hyperoxia

**Fig. 4:**
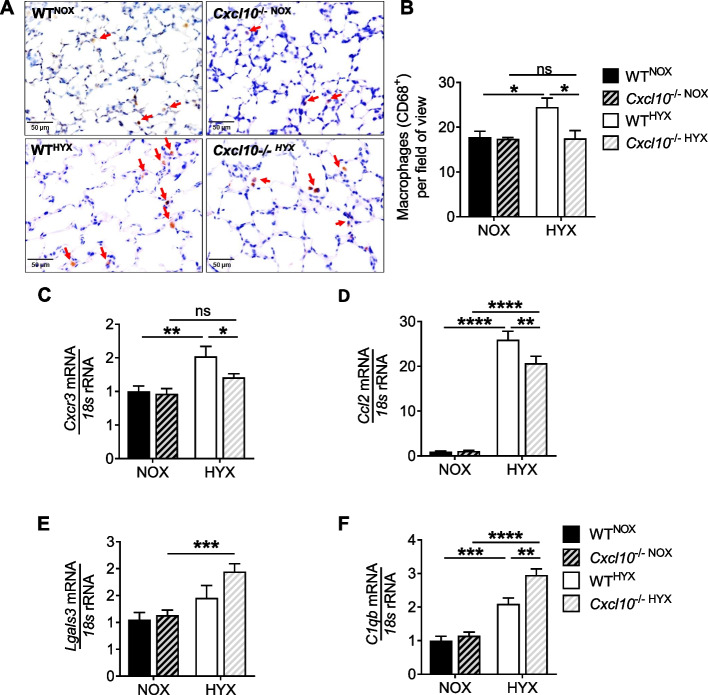
Deficiency of CXCL10 prevents the increase of macrophages in the lung. **A** Representative immunostaining of lung macrophage using CD68 as a marker. Red arrows indicate CD68^+^ stained cells in lungs exposed to normoxia (NOX, 21% O_2_) or hyperoxia (HYX, 85% O_2_) at postnatal day 14 (P14). **B** Quantification of CD68^+^ cells per field of view. **C**-**F** Assessment of mRNA expression of *Cxcr3* (**C**), *Ccl2* (C–C Motif Chemokine Ligand 2) (**D**), *Lgals3* (Galectin3) (**E**), and *C1qb* (complement C1q B chain) (**F**) in lungs at P14 after exposure to NOX or HYX. Mean ± SEM; *n* = 4–9/group. Two-way ANOVA: *p < 0.05; **p < 0.01; ***p < 0.001; ****p < 0.0001; ns = not significant; scale bar: 40X

Since CXCL10 acts as a chemoattractant for macrophages, we next tested if the loss of CXCL10 may reduce hyperoxia-induced macrophage influx to the lung. To this end, we performed immunohistochemical staining for CD68, a marker of active macrophages, and found a significantly increased number of macrophages in lungs of WT^HYX^ when compared to WT^NOX^. In contrast, *Cxcl10*^−/−^, however, were protected from macrophage influx in neonatal lungs after hyperoxia (Fig. 4A, B). Moreover, the expression of CXCL10 receptor *Cxcr3* increased in WT^HYX^ and was attenuated in *Cxcl10*^−/−HYX^ (Fig. 4C). Moreover, the mRNA expression of the CC-chemokine ligand 2 (*Ccl2*; monocyte chemotactic protein 1) was more than 20-fold increased in WT^HYX^ when compared to WT^NOX^, whereas loss of CXCL10 significantly reduced this effect (Fig. 4D). The expression of the M1-like macrophage marker *Tnfa*, but not *Il1b* was higher in *Cxcl10*^−/−HYX^ when compared to *Cxcl10*^−/−NOX^ or WT^HYX^ (Supplementary Fig. 1D, E). The gene expression of M2-like macrophages (*Arg1* and *Il4*) was neither affected in WT or *Cxcl10*^−/−^ exposed to normoxia or hyperoxia (Supplementary Fig. 1F, G). On the contrary, we found a significant higher mRNA expression of *Lgals3* and *C1qb* in *CXCL10*^−/−HYX^ when compared to WT^HYX^ (Fig. 4E, F). Galectin3 (*Lgals3*) and C1qb have been described to regulate migration/chemotaxis [31, 32] and to limit inflammasome activity [33], respectively. These results strengthened the notion that CXCL10 acts as a central hub of the immune response in neonatal lungs after hyperoxia through chemoattraction of macrophages with subsequent release of cytokines that can affect inflammation, cell survival and lung matrix remodeling.

### The beneficial effect of CXCL10 deficiency on alveolarization and lung matrix in lungs of neonatal mice exposed to hyperoxia persists after a regeneration phase

**Fig. 5:**
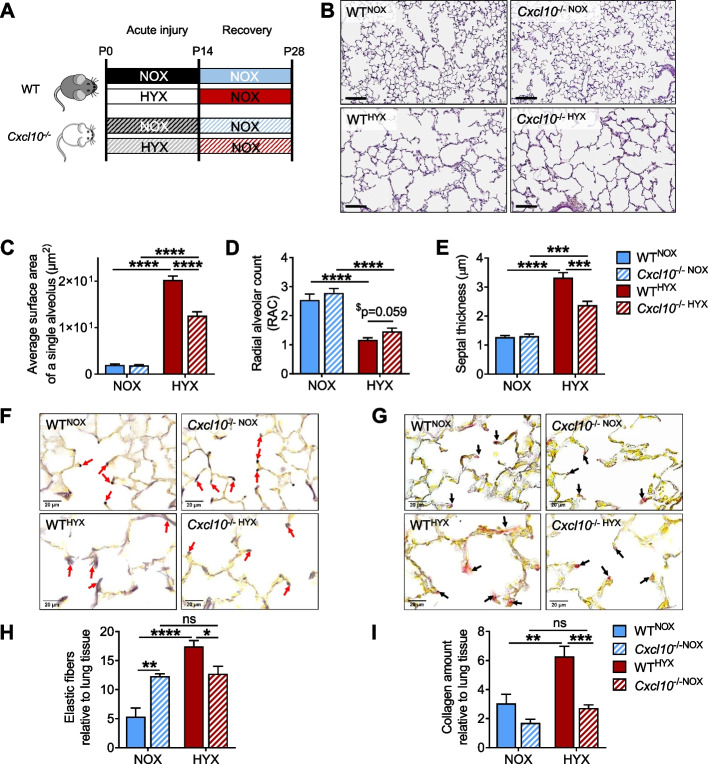
CXCL10 deficiency partially preserves alveolarization and lung matrix during regeneration from postnatal hyperoxia. **A** Experimental setup to study the role of CXCL10 in the regeneration phase after hyperoxia-induced lung injury: wildtype (WT) and *Cxcl10* knockout (*Cxcl10*^−/−^) mice were exposed to normoxia (NOX, 21% O_2_) or hyperoxia (HYX, 85% O_2_) from birth until postnatal day 14 (P14); from P14 to P28, all mice were exposed to NOX. **B** Representative images of hematoxylin and eosin (H&E) stained lung tissue sections in mice at P28. **C**-**E** Quantitative histomorphometric analyses for the average surface area of a single alveolus (**C**), radial alveolar count (RAC; **D**), and septal thickness (**E**). **F**-**I** Representative images showing the spatial distribution of elastic fibers (Hart’s stain; **F**) and collagen (Picro-Sirius red stain; **G**) in mouse lungs at P28. Quantitative analyses of the elastic fiber (**H**) and collagen content (**I**) relative to lung tissue in percentage (%; elastic fiber and collagen fraction). Mean ± SEM; *n* = 3–6/group; Unpaired t test: ^$^*p* < 0.05; Two-way ANOVA: *p < 0.05; **p < 0.01; ***p < 0.001; ****p < 0.0001; scale bar: 20X, 100X

The preceding findings demonstrate that CXCL10 is important in the hyperoxia-induced arrest of alveolarization through a regulation of macrophage invasion, the inflammatory response, cell survival, and lung matrix remodeling. However, the impact of CXCL10 during regeneration after neonatal hyperoxia has not been addressed to date. Therefore, we exposed WT and *Cxcl10*^−/−^ mice to either normoxia (21% O_2_) or hyperoxia (85% O_2_) from birth until P14, followed by recovery in normoxia for the next 14 days until P28 as shown in Fig. 5A. First, the quantitative histomorphometric analysis revealed a persistent increased average surface area of a single alveolus and a reduced RAC in WT^HYX^ when compared to WT^NOX^ at P28, indicating an irreversible arrest of alveolarization after hyperoxia. In contrast, loss of CXCL10 partially preserved alveolar formation as indicated by a lower average surface area of a single alveolus and an increased RAC in *Cxcl10*^−/−HYX^ when compared to WT^HYX^ (Fig. 5B-D). Next, we measured gene expression of collagens (*Col1a1*, *Col3a1*, and *Col4a4*) and did not detect any differences between WT^HYX^ and WT^NOX^. However, *Col1a1* was significantly reduced *Cxcl10*^−/−HYX^ when compared to *Cxcl10*^−/−NOX^. Similarly, *Col4a4* was lower in *Cxcl10*^−/−HYX^ than in WT^HYX^ (Supplementary Fig. 2A-C). Alveolar septae remained threefold thicker in WT^HYX^ than in WT^NOX^ even after recovery, whereas the septal thickness was attenuated by approximately 50% in *Cxcl10*^−/−HYX^ when compared to WT^HYX^ (Fig. 5E). To further substantiate the notion that changes in septal thickness is caused by lung matrix remodeling, we next assessed elastic fibers and collagen in the lungs. Interestingly, in contrast to P14, WT^HYX^ exhibited up to a 3-fold increase of elastic fibers relative to lung tissue in comparison to WT^NOX^ at P28, and the spatial distribution remained perturbed with “brush-like” structures. In *Cxcl10*^−/−HYX^, however, the elastic fiber fraction was attenuated when compared to WT^HYX^ and the spatial distribution remained at the secondary crests with less “brush-like” structures than WT^HYX^ (Fig. 5F, H). Similar to the findings in lungs after acute injury at P14, WT^HYX^ continued with a marked increase in collagen deposition relative to lung tissue when compared to WT^NOX^ at P28, whereas loss of CXCL10 fully protected from these effects (Fig. 5G, I). It is noteworthy, that *Cxcl10*^−/−NOX^ showed a significantly higher elastic fiber and lower collagen content than WT^NOX^ (Fig. 5H, I), further supporting the notion that CXCL10 plays a vital role in lung matrix remodeling, not only during acute injury, but also during regeneration.

### Loss of CXCL10 limits macrophage influx into the lung during regeneration from neonatal hyperoxia

**Fig. 6:**
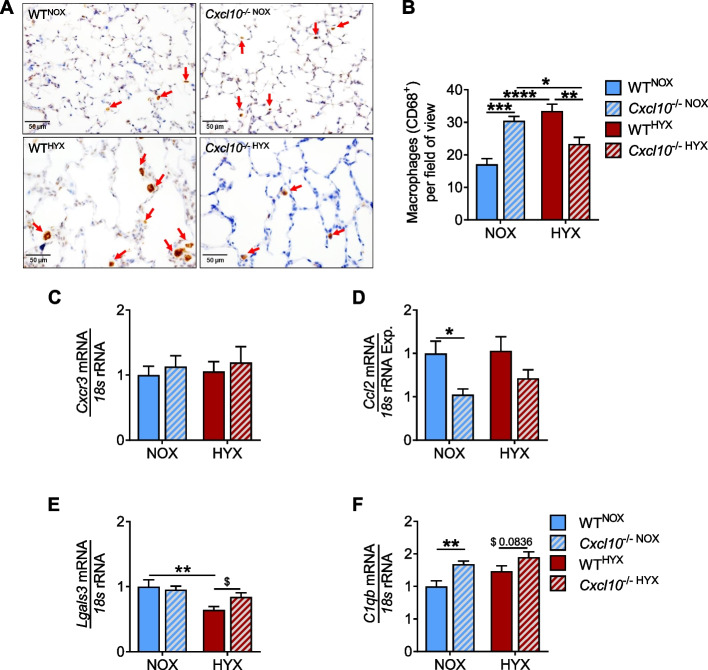
CXCL10 deficiency limits the macrophage influx to the lung during the regeneration phase after postnatal hyperoxia. Experimental design: wildtype (WT) and *Cxcl10* knockout (*Cxcl10*^−/−^) mice were exposed to normoxia (NOX, 21% O_2_) or hyperoxia (HYX, 85% O_2_) from birth until postnatal day 14 (P14); from P14 to P28, all mice were exposed to normoxia. **A**, **B** Representative immunostaining of macrophages using CD68 as a marker. Red arrows depict CD68^+^ stained cells (**A**). Quantification of CD68^+^ cells in the lung per field of view (**B**). **C**, **D** Measurement of mRNA expression of *Cxcr3* (**C**), *Ccl2* (C–C Motif Chemokine Ligand 2) (**D**), *Lgals3* (Galectin3) (**E**), and *C1qb* (complement C1q B chain) (**F**) in lungs at P28; *18 s* rRNA served as housekeeping gene. Mean ± SEM; *n* = 4–9/group; Unpaired t test: ^$^*p* < 0.05; Two-way ANOVA: *p < 0.05; **p < 0.01; ***p < 0.001; ****p < 0.0001; scale bar: 40X

Activated macrophages regulate lung matrix remodeling that can adversely affect alveolar development. Thus, preventing the migration of macrophages to the lung could not only preserve normal lung matrix synthesis and assembly but thereby also promote alveolar growth. We assessed the number of activated macrophages in the lungs after recovery from neonatal hyperoxia at P28 using immunohistochemical staining for CD68 and found an almost 2-fold increase of macrophages in WT^HYX^ when compared to WT^NOX^. *Cxcl10*^−/−HYX^, however, were significantly protected from the increase of macrophages during regeneration after neonatal hyperoxia when compared to WT^HYX^ (Fig. 6A, B). The increase of macrophage invasion in WT^HYX^ was related to a persistent increase of *Cxcl10* expression in lungs at P28 after recovery from HYX (Supplementary Fig. 3). Interestingly, in contrast to P14, we did not determine changes in the gene expression of *Cxcr3*, the CXCL10 receptor, in WT or *Cxcl10*^−/−^ at P28 after neonatal hyperoxia. (Fig. 6C). However, the mRNA expression of *Ccl2* was significantly reduced in *Cxcl10*^−/−NOX^ and by trend in *Cxcl10*^−/−HYX^ when compared to WT^NOX^ and WT^HYX^, respectively (Fig. 6D). Moreover, we assessed pro-inflammatory markers and found a significant reduction of *Tnfa* by 50% in *Cxcl10*^−/−HYX^ when compared to WT^HYX^. Similarly *Il1b* mRNA was markedly lower in in *Cxcl10*^−/−HYX^ than in *Cxcl10*^−/−NOX^. On the other hand, M2-like macrophage markers (*Arg1*, *Il4*) were not altered by hyperoxia or *Cxcl10*^−/−^ (Supplementary Fig. 4A-D). Interestingly, *Lgals3* was significantly downregulated in WT^HYX^ when compared to WT^NOX^, whereas this effect was attenuated in *Cxcl10*^−/−HYX^. *Lgals3* encodes for Galectin3, a protein that limits invasion of immune cell; a loss of Galectin3 expression as seen in WT^HYX^ but not in *Cxcl10*^−/−HYX^ could partly explain increased macrophage invasion in WT but not *Cxcl10*^−/−^ after hyperoxia (Fig. 6E). Next, we measured the gene expression of *C1qb,* encoding for a protein with anti-inflammasome function, and found a significant increase in *Cxcl10*^−/−NOX^ when compared to WT^NOX^ lungs (Fig. 6F). Our data indicate that CXCL10 is a key regulator of macrophage function, and its effect is dependent on the microenvironment and exposome. While loss of CXCL10 promotes macrophage invasion to the lung under normoxia, the macrophages do not seem to be polarized towards a pro-inflammatory sub-type. Conversely, knockout of CXCL10 under hyperoxia results in protection from invasion, suggesting a microenvironment-modulated CXCL10-dependent differentiation of macrophages.

### Hyperoxia induces *Cxcl10* that drives migration of human and murine macrophages through its receptor CXCR3

**Fig. 7:**
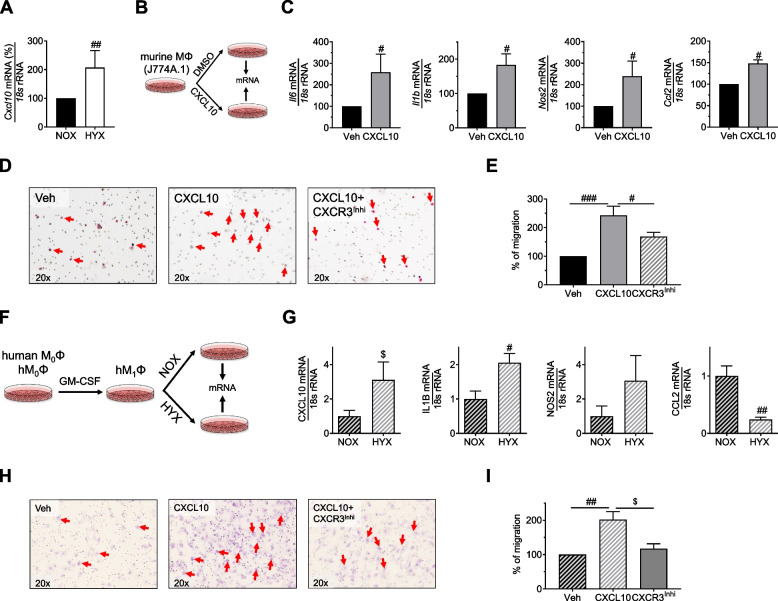
Inhibition of the CXCR3-CXCL10 axis limits migration of cultured mouse and primary human macrophages. **A** Murine macrophages (J774A.1) were cultured in a serum-rich medium until the cells were 80% confluent, followed by 12 h in serum-reduced medium and then 12 h in serum-rich medium. Afterward, the cells were exposed to normoxia (21% O_2_, NOX) or hyperoxia (85% O_2_) for 24 h. Measurement of *Cxcl10* mRNA using quantitative RT-PCR; *18 s* rRNA served as a housekeeping gene. **B**, **C** Experimental setup to determine the functional role of CXCL10 in macrophage differentiation (**B**). Assessment of mRNA expression of *Il6* (interleukin 6), *Il1β* (interleukin 1beta), *Nos2* (nitric oxide synthase), and *Ccl2* in macrophages treated with CXCL10 (10 ng/ml) or vehicle for 48 h; *18 s* rRNA served as a housekeeping gene. **D**, **E** Representative images of migrated J774A.1 macrophages treated with either only CXCL10 (10 ng/ml) or CXCL10 plus CXCR3 antagonist (300 nm/ml) for 24 h; controls were treated with DMSO (**D**). Quantification of migrated macrophages per field of view (20X) (**E**). **F**, **G** Experimental plan to study the impact of HYX on differentiation of primary human M1-like macrophages: human M0 macrophages were differentiated into M1-like macrophages by treatment with GM-CSF (100 ng/ml) for 7 days, followed by exposure to NOX or HYX (**F**). Assessment of CXCL10, IL1B, NOS2, and CCL2 expression in human M1-like macrophages; *18 s* rRNA served as housekeeping gene (**G**). **H**, **I** Representative images of migrated human M0-like macrophages treated with either only CXCL10 (10 ng/ml) or CXCL10 plus CXCR3 antagonist (300 nm/ml) for 24 h; controls were treated with DMSO (**H**). Quantification of migrated human M0-like macrophages per field of view (20X) (**I**). Mean ± SEM; *n* = 4–9/group; Mann–Whitney test: #*p* < 0.05; ##*p* < 0.01; ###*p* < 0.001, Paired t-test: $ *p* < 0.05; scale bar 20X

Hyperoxia significantly induced mRNA expression of *Cxcl10* in mouse peritoneal macrophages (J774A.1; mMΦ), confirming the impact of increased oxygen concentration on CXCL10 signaling (Fig. 7A). To further understand the possible role of a CXCL10-CXCR3 axis in the regulation of macrophage function, we first treated J774A.1 with CXCL10 (10 ng/ml) or DMSO as vehicle control for 48 h in serum-reduced medium, followed by assessment of gene expression using qRT-PCR. Stimulation with CXCL10 caused an almost 2-fold induction of *Il6*, *Il1b*, *Nos2* (iNOS), and *Ccl2*, indicating an M1-like activation of murine macrophages (Fig. 7B, C). For the migration assay, we used the Boyden chamber assay and stimulated J7741.A with either CXCL10 (10 ng/l) [lower chamber with 1% FBS medium] alone or in combination with CXCR3 antagonist (300 nM/ml) [upper chamber with serum-reduced medium] for 24 h; DMSO served as vehicle control. Stimulation with CXCL10 induced a 2-fold increase in the migration rate of murine macrophages, whereas blocking the CXCR3 receptor significantly reduced the CXCL10-induced migration of macrophages (Fig. 7D, E). Treatment with the CXCR3 receptor antagonist alone did not affect murine macrophage migration (Supplementary Fig. 5A, B). To translate our data to human, we next used human blood monocyte-derived macrophages. Since prior studies from our group demonstrated that hyperoxia causes M1-like polarization in mouse lungs [8], we now polarized human macrophages towards M1-like phenotype by treatment with GM-CSF (100 ng/ml for 7 days with media change at alternating days) (hM1Φ; Fig. 7F). Similar to mice, exposure of hM1Φ to hyperoxia (85% O_2_) for 48 h induced gene expression of CXCL10 and IL1B, whereas CCL2 was downregulated (Fig. 7G). In analogy to the mMΦ, we treated hM0Φ with either CXCL10 (10 ng/ml) alone or in combination with CXCR3 antagonist (300 nM/ml) for 24 h; DMSO served as vehicle control. The analysis of the migration assay revealed a significant increase in hM0Φ migration that was prevented by blocking the CXCR3 receptor (Fig. 7H, I); CXCR3 receptor blocker alone did not have a marked effect on macrophage migration (Supplementary Fig. 5C, D). These data do not only demonstrate that hyperoxia induces *Cxcl10* expression, but also that CXCL10 further triggers M1-like inflammatory phenotype and promotes migration of macrophages.

### Macrophage (CD68^+^) influx with elevated CXCL10 expression in lungs of infants with BPD

**Fig. 8:**
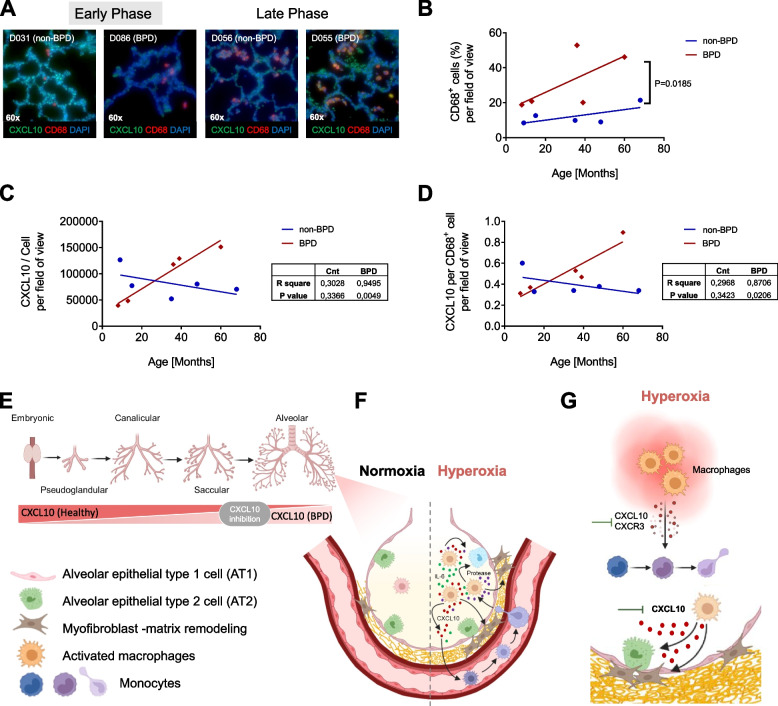
Temporal dynamics of macrophage-specific CXCL10 expression in lungs of infants with or without bronchopulmonary dysplasia (BPD). **A** Representative co-localization of CD68 (red, immunofluorescent staining) with CXCL10 (green; in situ hybridization) in age-matched lungs of infants with and without BPD (Cnt vs BPD). **B-D** Linear regression analyses of the number of macrophages (MΦ; CD68^+^ cells; **B**), CXCL10 mRNA per cell and field of view (**C**), and CXCL10 mRNA per MΦ (CD68^+^ cells) and field of view (**D**) related to age (lungs of infants with BPD: red; lungs of infants without BPD, non-BPD: blue); r2 and *p*-value are indicated next to the respective graph; n = 5 per group; scale bar: 60X. **E**–**G** A proposed working model of the functional role of hyperoxia-related macrophage-derived CXCL10 in the pathogenesis of BPD: decreasing expression of CXCL10 during postnatal lung development in healthy lungs that is disrupted by prolonged hyperoxia, thus suggesting CXCL10 inhibition as a therapeutic strategy (**E**). Hyperoxia promotes the differentiation of macrophages towards a M1-like inflammatory phenotype with expression of IL-6 and CXCL10, and an imbalance of protease and proteolytic activity. This cytokine storm (e.g., IL-6 and CXCL10) together with elevated protease activity negatively affects the survival of alveolar epithelial type 2 cells (AT2), activates fibroblasts, and causes lung matrix remodeling (**F**). The release of CXCL10 from hyperoxia-activated macrophages adversely affects the regenerative alveolar niche and spatial distribution of elastic fibers and collagen. In contrast, CXCL10 deficiency protects from these changes and pharmacological inhibition of CXCR3 reduces the macrophage migration, offering a new therapeutic target to treat BPD

Finally, we analyzed the lungs of age-matched infants with or without BPD (Table 1). To this end, we first performed immunofluorescent staining for CD68 (marker of activated macrophages) combined with in situ hybridization of CXCL10. Lungs with BPD showed a significant increase of CD68^+^ cells (macrophages) per field of view with age when compared to non-BPD lungs (*p* = 0.0185) (Fig. 8A, B). Subsequent quantification showed a decrease in the intensity of CXCL10 per DAPI^+^ cell in non-BPD lungs with age, whereas the intensity of CXCL10 per DAPI^+^ cell increased significantly in BPD lungs with age (*R*^2^ = 0.9495; *p* = 0.0049) (Fig. 8C). In addition, the intensity of CXCL10 per CD68^+^ cell was significantly higher in lungs with BPD than in non-BPD lungs with age (*R*^2^ = 0.8706; *p* = 0.0206) (Fig. 8D). In summary, these findings support the hypothesis that hyperoxia-induced activation of CXCL10 signaling in M1-like macrophages could contribute to the progression of BPD over time.

**Table 1:** Clinical data of lungs of infants with and without bronchopulmonary dysplasia (BPD)

**Healthy human sample**				
**Donor ID**	**Calculated age (months)**	**Sex**	**Race**	**ClinPathDx: control**
D031	7.64	Male	White	Normal growth and structure
D090	14.70	Female	NR	Normal growth and structure
D046	36.5	Male	White	Normal growth and development
D139	47.67	Female	One race	Normal lung growth and structure
D056	68.21	Male	White	Normal growth and development
**BPD human sample**				
**Donor ID**	**Calculated age (months)**	**Sex**	**Race**	**ClinPathDx: BPD/CLD**
D086	8.94	Male	White	25 weeks at birth, probable BPD with respiratory failure
D141	13.40	Male	White	25 weeks at birth, BPD; moderate deficient alveolarization, chronic inflammation, airway muscle hyperplasia, medial hypertrophy of small arteries
D039	39.13	Female	NR	23 weeks at birth, “new” BPD
D053	37.87	Male	White	24 weeks at birth, BPD, mild-moderate bronchiolitis
D055	59.96	Male	White	32 weeks at birth, possible mild BPD, asthma, moderate bronchopneumonia

*NR* not reported, *CLD* chronic lung disease, *ClinPathDx* clinical pathology diagnostic

## Discussion

The present study investigates the role of the CXCL10-CXCR3 signaling in the inflammatory response and macrophage influx in experimental and clinical BPD. In a murine model of hyperoxia-induced neonatal lung injury, transcriptome analysis identified CXCL10 as a central hub in lung inflammation of neonatal mice exposed to hyperoxia. Neonatal *Cxcl10* null mice were protected from (i) macrophage influx to the lung, (ii) lung matrix remodeling with increased collagen deposition and perturbed spatial distribution of elastic fibers as well as (iii) lung cell apoptosis and loss of AT2, (iv) thereby preserving alveolar formation during hyperoxia-induced acute lung injury. Analysis of murine and human macrophages revealed a hyperoxia-induced M1-like differentiation and a CXCR3-mediated macrophage migration. Finally, our data demonstrate an increased CXCL10 expression in macrophages over time in the lungs of infants with BPD *vs* non-BPD, offering CXCL10-CXCR3 as a potential target for inflammation-driven progression of BPD.

Inflammation is important in the pathogenesis of BPD [9, 34, 35]. In particular, macrophages accumulate in the lungs of infants with BPD as well as in hyperoxia-induced lung injury as a model of BPD [8]. While recent studies report multiple macrophage subtypes along with diverse immunomodulatory functions in the developing lung [36] as well as after neonatal hyperoxia [9], the mechanisms regulating macrophage homeostasis and activation remain poorly understood. Prior studies from our group showed a macrophage influx to the lung and a differentiation towards pro-inflammatory (M1-like) phenotype in neonatal mouse lungs after hyperoxia, resulting in a reduced alveolarization and an excessive epithelial cell apoptosis [8]. Our current transcriptomic approach identified CXCL10 to be central in the lung inflammatory network in neonatal mice after hyperoxia. The clinical relevance of CXCL10 was further supported by clinical studies in which elevated concentrations of CXCL10 in tracheal aspirates were associated with evolving BPD or death in premature infants [37]. In the present study, we examined the time course of CXCL10 expression in lungs of non-BPD and BPD infants. While the CXCL10 expression was high at the early stage in non-BPD lungs and decreased over time, we detected an opposite trend with a significant correlation of increasing CXCL10 expression in BPD lungs with age. Of note, this finding was associated with a higher number of macrophages as well as increased CXCL10 per macrophage in BPD when compared to non-BPD lungs. CXCL10 as a macrophage-regulating chemokine may contribute to the migration of immune cells, inflammation, and clinical course of BPD, and could thereby serve as a biomarker of BPD in very premature infants.

This notion was further supported by our experimental BPD model, where we associated hyperoxia-induced *Cxcl10* with macrophage infiltration to the lung. In contrast, *Cxcl10* null mice were protected from macrophage influx, resulting in an attenuated lung growth arrest. Various studies pointed out the role of CXCL10 as a chemokine that plays a critical role in the migration of immune cells, particularly T cells, NK cells, and macrophages [22, 23, 38]. Migration of immune cells is induced by the binding of CXCL10 to its receptor, CXCR3 that is expressed on the surface of T cells, NK cells, and macrophages, resulting in macrophage migration towards the site of CXCL10 production [22, 23]. Here, we demonstrate that exposure of cultured macrophages to hyperoxia increases the expression of CXCL10, which in turn increases mRNA expression of *Il6*, *Il1b*, *Nos2*, and *Ccl2* in murine macrophages. CXCL10 signals through the CXCR3 receptor and thereby regulates the infiltration of immune cells and macrophage homeostasis in the tissue [23, 39–41]. Our in vitro study shows that CXCL10 drives migration of murine and human macrophages significantly, a process that is inhibited by the CXCR3 antagonist. However, other immune and epithelial cells produce different chemokines and may be involved in chemoattraction in the pathogenesis of the BPD as well [42]. Our in vitro data combined with the in vivo findings indicate a migratory and immunomodulatory role of CXCL10 in hyperoxia-induced neonatal lung injury and BPD, offering a possible target for pharmacological treatment or prevention.

Inflammation and macrophage activation can contribute to lung matrix remodeling in BPD through an interplay with other cell types, e.g., fibroblasts and epithelial cells [8, 43–45]. For example, reports demonstrate an important role of the imbalance in macrophage activation in the initiation of fibrotic remodeling in lungs with idiopathic pulmonary fibrosis (IPF). Moreover, a recent study showing a pro-fibrotic macrophage response in SARS-CoV2 infection points out the plasticity of macrophages and their important role in matrix remodeling and lung repair [14]. Similarly, targeting subtypes of pro-fibrotic macrophages or targeting CXCR3 limits the progression of fibrosis [46]. Collectively, these studies shed light on the role of macrophages in altering ECM composition and deposition. Studies to determine cell–cell communication between macrophages and fibroblasts could help to understand how certain signals promote fibrotic changes and differentiation of subsets of mesenchymal cell populations. For example, the CXCR3-CXCL10 axis modulates the immune cell proportion in tissue in part through the regulation of infiltration, e.g., inhibiting NK cells and anti-tumor T cells [29, 47] or promoting neutrophils and macrophages [38, 40]. An imbalance in immune cell proportion can favor a microenvironment for fibrosis-related tissue remodeling [47]. Moreover, CXCL10 secreted by activated fibroblasts plays a crucial role in triggering M1-like polarization of macrophages in an inflammatory environment [24]. It also is involved in the regulation of inflammation and immune responses, which can affect the disease progression [37, 48, 49]. Here, we observed an increased number of macrophages in the lungs of *Cxcl10* null exposed to room air. The mechanisms by which CXCL10 interferes with the macrophage infiltration under physiological conditions remain unclear but could be related to an imbalance in chemoattractant chemokines and distinct polarization of macrophages. Moreover, our data demonstrate that the functional impact of CXCL10 on the expression of macrophage markers depends on the micro-environment such as the oxygen concentration. While loss of CXCL10 itself promotes macrophage invasion to the lung under normoxia, they do not seem to be polarized towards a pro-inflammatory sub-type. Conversely, knockout of CXCL10 under hyperoxia results in protection from invasion, possibly by regulating *Lgals3* (Galectin3) and *C1qb* that limits immune cell invasion and inflammasome activity, respectively [31–33]. This immunomodulatory function of CXCL10 may underlie the reduced increase in septal thickness and deposition of collagen in the alveolar septa and thus may also provide an advantage for alveolar structure and function during regeneration. Our current findings suggest a distinct CXCL10-mediated differentiation of macrophages depending on oxygen concentration. Further studies are required to explore the role of CXCL10 in macrophage homeostasis and migration in healthy and different disease conditions.

CXCL10 is associated with the pathogenesis of lung diseases, including BPD and IPF [37, 50]. In contrast, experimental studies show that blocking CXCL10 has therapeutic potential [40]. The present study did not only focus on the role of CXCL10 in acute neonatal lung injury after hyperoxia, but also studied the impact of CXCL10 on lungs during the recovery from postnatal hyperoxia. We demonstrate that the simplified alveolar structure after neonatal hyperoxia persists beyond the regeneration phase and is associated with irreversible changes in the lung matrix over time. The loss of CXCL10, however, increased the lung proteolytic activity (MMP9) and inhibited fibrotic TGFβ-SMAD2 signaling, which was associated with protection of the lung from collagen deposition and perturbed elastic fiber assembly during the acute injury, but in particular during the regeneration phase. These effects could be in part mediated by a blockade of macrophage influx to lungs in neonatal mice with *Cxcl10* knockout. The possible relationship between fibro angiogenetic changes and macrophage migration as well as activation has been previously reported [23, 39–41] and might contribute to hyperoxia-induced lung injury. Since we did not find marked changes in the gene expression of collagens in the lungs after hyperoxia, the protective effect of the *Cxcl10*^−/−^ on lung matrix remodeling could be likely related to other interrelated mechanisms, including attenuation of pro-fibrotic signaling pathways or balancing of proteolytic processes and degradation of lung matrix. In the present study, the knockout of *Cxcl10* attenuates the activation of TGFβ signaling (Fig. 3H, I) and increases the proteolytic activity of MMP9. Both Cxcl10-dependent effects could change the balance of matrix synthesis, deposition, and degradation after hyperoxia favoring anti-fibrotic effects.

## Conclusion

In summary, our data identified CXCL10-CXCR3 signaling to be central in the pathogenesis of hyperoxia-induced neonatal lung injury as a model of BPD and in lungs of infants with BPD. We demonstrate that hyperoxia-induced CXCL10 promotes macrophage infiltration to murine lungs possibly through CXCR3, drives collagen deposition, perturbs elastic fiber formation and positioning, causes lung cell apoptosis, and ultimately leads to reduced alveolar formation. In contrast, CXCL10 deficiency protects neonatal lungs from macrophage influx, lung matrix remodeling, and loss of AT2, enabling thereby lung growth after hyperoxia (Fig. 8 E–G). Since we show macrophage influx with increased CXCL10 expression in lungs of infants with progressive BPD, blocking CXCR3-CXCL10 could offer a new avenue to treat infants at risk for BPD and ameliorate the course of BPD.

## Material and methods

### Sub-analysis of our publicly available RNA sequencing (RNA-Seq) data set

For transcriptomic analysis, we used an RNA-Seq dataset of lungs of newborn mice exposed to normoxia (21% O_2_, NOX) or hyperoxia (80% O_2_, HYX) from birth until P14. This RNA-Seq dataset was previously published by our group and is deposited and publicly available in Dryad repository (https://doi.org/10.5061/dryad.rr4xgxd8m). Genes with *p* < 0.05 and fold change > 1.5-fold were considered differentially expressed genes (DEG). In the present study, first, we generated a heatmap of the 25 most up- and down-regulated DEG based on their fold change using heat mapper (http://heatmapper.ca/) [51]. For subsequent data analysis we used http://www.bioinformatics.com.cn/srplot, a free online platform for data analysis and visualization. We performed KEGG pathway annotation analysis based on the enrichment score using SRPLOT [52, 53]. For studying the inflammatory network, DEGs associated with the top-ten most up- or downregulated KEGG pathways were used as input for gene ontology (GO) analysis. Visualization of the interaction of the DEGs with the pathways based on GO annotation was performed with a sneaky plot [54, 55].

### Animal studies and ethical approval

All animal studies were approved by the local government authorities (LANUV, NRW, Germany; 87–51.04.2010.A372; 84–02.04.2015.A120; 84–02.04.2020.A95). Adult and neonatal male and female C57BL/6 J (WT), as well as B6.129S4-Cxcl10tm1Adl/J (Cxcl10^−/−^; Jackson laboratory; strain #: 006087) mice, were housed in humidity- and temperature-controlled rooms exposed to a 12 h dark/light cycle and were allowed food and water ad libitum.

### Neonatal hyperoxia-induced lung injury model

Newborn mice were exposed to hyperoxia as described previously [56]. In brief, newborn mice were pooled and randomized to dams on the day of birth (born within 12 h of each other). Half of the litters were exposed to 85% O_2_ (hyperoxia, HYX), whereas the other pups were at room air [21% O_2_; normoxia, NOX]. The dams were rotated between hyperoxia and normoxia daily. Neonatal mice were exposed to hyperoxia in a 90 × 42 × 38 cm plexiglas chamber and the oxygen concentration was monitored with a Miniox II monitor (ProOx 360, Biospherix, USA). The study design comprised two sets of experiments: (i) neonatal mice were exposed to hyperoxia or normoxia from birth until postnatal day 14 (P14); and (ii) after exposure to hyperoxia from birth until P14, mice were transferred to normoxia for regeneration until P28; the control mice were in normoxia from birth until P28. At the end of both time points (P14 and P28), lungs were excised and studied.

### Tissue preparation

We sacrificed the mice and excised the lungs at P14 and P28 as previously described [56–58]. After ligation of the right bronchus, the right lung was excised and immediately snap-frozen and stored at -80 °C for molecular assessment; the left lung was pressure-fixed with 4% paraformaldehyde (PFA) in phosphate-buffered saline (PBS), followed by paraffin embedding as described in prior studies from our group [57].

### Quantitative histomorphometric analysis

PFA-fixed and paraffin-embedded lung sections were used for quantitative histomorphometric analysis as previously described by [8]. Briefly, Isotropic Uniform Random (IUR) sectioning was performed to obtain 3 µm lung sections. Afterwards, four tissue sections were randomly chosen for hematoxylin and eosin (H&E) staining [59]. For imaging, the stained lung sections were scanned with a slide scanner (Leica SCN400 Slide Scanner, Houston, USA), followed by measurement of histomorphometric parameters in up to ten fields of view per lung Sect. (20x) as described previously [8]: radial alveolar count (RAC), average surface area of a single alveolus, and septal thickness. Fields of view were not analyzed when airways or vessels covered approximately more than 15% or lungs were atelectatic or not well inflated.

### RNA isolation and quantitative reverse transcriptase PCR (RT-PCR)

Lung tissue, mouse ascites macrophages (J774A.1), and human primary macrophages were used for RNA extraction as described previously [56]. For subsequent cDNA synthesis, 1 μg of mRNA of each sample was used. For measurement of gene expression, quantitative RT-PCR (qRT-PCR) was performed in 96 well plates (FrameStar, 96 well plate #4ti-0770/C, UK) with DNA-DYE (GoTaq® qPCR Master Mix, #A600A, Madison, USA or Platinum™ Quantitative PCR SuperMix-UDG w/ROX, #11743500, Netherlands) using a 7500 Real-Time PCR System (Applied BiosystemsTM, Foster City, USA) as previously described [59]. The Primers were designed using Primer Express Software 3.0 (Applied BiosystemsTM, Foster City, USA) and sequences are listed in Supplementary Table 2. Gene expression was quantified based on the ΔΔCt-method and expressed as fold induction of mRNA expression. The housekeeping gene 18 s rRNA was used to normalize genes of interest.

### Immunoblot

Proteins were isolated from snap-frozen lungs and the concentration was measured for immunoblots as described previously [59]. In brief, a concentration of 20 µg of protein per sample was separated using SDS-PAGE and transferred onto a nitrocellulose membrane. Afterwards, the membrane was blocked with 5% milk (Roth, #T145.3, Germany) and 2% bovine serum albumin (BSA; Roth, # 8076.3, Germany) in TBS-Tween (0.1% Tween®20; Sigma Aldrich, #P1379, Germany). Next, the membrane was probed with primary antibodies at 4° C overnight: rabbit anti-phosphorylated SMAD2 (pSMAD2 (Ser465/467; 138D4; Cell Signaling, # 3108, Danvers, USA, 1:1000), rabbit anti-total SMAD2 (D43B4; Cell Signaling, # 5339, Danvers, USA, 1:1000), mouse anti-β-ACTIN (Cell Signaling, #3700, Danvers, USA, 1:10,000). Next day, after washing, horseradish-peroxidase (HRP)-linked secondary antibody (anti-mouse IgG, HRP-linked antibody, #7076 or anti-rabbit IgG, HRP-linked antibody, #7074, Cell Signaling, Danvers, MA, USA) was applied at room temperature (RT) for 1 h. Protein bands were visualized using ChemiDoc XRS + Imaging System (Bio-Rad Laboratories GmbH, Germany). Densitometric analysis was performed using Image Lab software 5.2.1 (Bio-Rad Laboratories GmbH, Germany) to quantify protein amounts as previously described [57].

### CXCL10 ELISA

As described previously serum samples were collected and protein concentration was measured and used for assessment of CXCL10 serum concentration using commercially available ELISA kit. The ELISA was performed according to the manufacturer’s guidance (Mouse IP-10, CXCL10, SimpleStep ELISA® Kit, Abcam, #ab214563). In brief, the ELISA plate was incubated with CXCL10 capture antibody at 4 °C overnight; afterwards, a 100 μl protein sample was added per well and incubated at RT for 2 h. The wells were then washed with washing buffer and 100 μl of the detection antibody was added to each well and incubated at RT for 1 h. Next, each well was washed and exposed to 100 μl of avidin-HRP conjugated antibody for 30 min at RT. Finally, after washing, additional 100 μl of TMB solution (substrate for avidin-HRP) was added per well and incubated for 15 min; the reaction was then stopped by the stop solution (2N H_2_SO_4_). In the end, the optical density (OD) was measured at 450/570 nm (Tecan Infinite® 200 PRO, Switzerland).

### Zymography for detection of matrix metalloproteinase 2 (MMP2) and MMP9

Proteins from total lung homogenates were isolated in PBS, concentration was measured, and 30 μg protein per sample was used for zymography as previously described [59]. Briefly, first, the proteins were incubated with the 3.5X loading buffer (0,22 M TRIS pH 6,8; 35% Glycerol; 7% SDS; Bromphenolblue) for 10 min at RT, followed by protein separation using SDS-PAGE gel. The separation gel was supplemented with 2 mg/ml gelatin (G2500 Sigma-Aldrich, Germany) as a substrate for MMP2 and MMP9. After the protein separation, the gel was incubated in 1X renaturation buffer (25% TritonX-100) for 30 min at RT. Next, the gel was treated with the development buffer (0,5 M TRIS; 2 M NaCl; 50 nM CaCl_2_; 0,2% Brij-35; pH 8,0) for 30 min at RT, and then overnight at 37 °C. To visualize the degradation of the gelatin as an indicator of MMP2 and MMP9 activity during development, the gel was then incubated with Coomassie staining solution (0,1% Coomassie Brilliant Blue; 25% Isopropanol; 10% acetic acid) for 30 min at RT. Excess Coomassie staining solution was removed by washing in dH_2_O. The bands of degraded gelatin were determined with UV transillumination using a ChemiDoc XRS + Imaging System and Image Lab™ software (Bio-Rad Laboratories GmbH, Germany). The bands were analyzed by densitometry. For this purpose, the program Image Lab™ was used to generate a negative image of the zymogram, after which the band density was measured.

### TUNEL and SFTPC immunofluorescent staining

Co-immunofluorescent staining was performed as previously described [8]. In brief, tissue sections were deparaffinized and rehydrated, followed by antigen retrieval with proteinase K solution (Thermo Scientific™, #EO0491, Waltham, MA, USA) for 15 min at 37 °C. Using Sea Block (Thermo Scientific™, #37527, Waltham, MA, USA), tissue sections were blocked. Then, the lung sections were incubated with terminal uridine deoxynucleotidyl transferase dUTP terminal nick end labeling (TUNEL) solution as per the manufacturer’s instructions (In Situ Cell Death Detection Kit, Roche, #11684795910, Germany) for 1 h at 37 °C. Subsequently, the sections were washed with PBS-T and incubated with rabbit anti-pro-SFTPC antibody (Merck, #AB3786, Germany, 1:200) in dark at 4 °C overnight. The next day, the sections were washed with PBS-T and treated with a secondary antibody conjugated with CY3 (Dianova, #111–165-003, USA) for 1 h at RT; cell nuclei were stained with DAPI (Sigma-Aldrich, #D9542, Germany). Finally, the tissue sections were mounted and images were taken directly at 40 × and 100 × magnification using a fluorescence microscope (Olympus, Hamburg, Germany). Images were used to calculate the total TUNEL^+^ and SFTPC^+^ cells in four fields of view per animal.

### Immunohistochemical staining

Tissue sections were deparaffinized and rehydrated as previously described [8]. Afterwards, antigen retrieval was performed by cooking slides with 10 mM Citrate buffer pH 6 (Dako, Cat. No. S2369, Germany) at 99 °C for 25 min. Next, the tissue sections were treated with a blocking solution (Sea Block, Thermo Scientific™, Cat. No. 37527, Netherlands) at RT for 1 h. Slides were incubated overnight with the primary antibody rabbit anti-CD68 (Abcam, Cat. No. ab125212, UK, 1:200) at 4 °C. Next, the slides were washed with PBS and then exposed to the secondary antibody (Histofine® MOUSESTAIN KIT, Nichirei, #414341F, Japan, for rabbit primary antibody) for 1 h at RT, followed by mounting using Neo-Mount® (Merck, #109,016, Germany). The whole tissue section was scanned with a slide scanner (Leica SCN400 Slide Scanner, Houston, USA). CD68^+^ cells (macrophages) were counted in up to ten fields of view per slide and randomly selected lung sections per animal from 5–7 animals per group were analyzed.

### Picro Sirius-Red staining

Lung matrix remodeling, including collagen deposition and fibrotic processes, was analyzed using Picro Sirius-Red staining. First, sections were deparaffinized, rehydrated and washed in distilled water. Then, they were immersed for 4 min in a 0.2% phosphomolybdic acid solution and then stained for 75 min with a Picro Sirius-Red solution as described previously [59]. After washing twice in 0.5% acetic acid, the sections were dehydrated with EtOH [2 × 100% (vol/vol) EtOH] and clarified in Neo-Clear to be subsequently covered. Imaging of the tissue section was performed with a slide scanner (Leica SCN400 Slide Scanner, Houston, USA). Two to four sections per animal were used for staining and up to 6 fields of view per section were analyzed.

### Hart’s elastin stain

Lung sections were stained for elastic fibers (purple) using Resorcin Fuchsin from Weigert (Weigert’s Iron Resorcin and Fuchsin Solution; Carl Roth, X877.3) and counterstained yellow with Tartrazine [0.5% in 0.25% acetic acid (Dianova, cat. no. TZQ999, USA)]. After washing with PBS, slides were dehydrated with EtOH and slides were covered with mounting media.

### Semi-quantitative analysis of lung collagen and elastic fiber content

Quantification of the collagen and elastic fiber content in the lung tissue was performed with ImageJ [60]. For this purpose, a light microscope (BX43; Olympus, Germany) was used; after white-balance, images of up to ten fields of view at 20 × magnification per lung section were taken applying a constant exposure time. We next set a uniform color threshold for the ImageJ2 software [61] to differentiate between elastic fibers (Hart’s stain) or collagen (Picro Sirius-Red stain) and lung tissue (Hue: 160–255; Saturation: 31–255; Brightness: 70–255). First, images were converted to RGB, and blue color images were selected for further analysis. Next, the threshold was set to cover the whole lung tissue area in the field of view; this value was considered the threshold value. The positive-stained area was defined manually as elastic fibers or collagen; next, the total elastic fiber or collagen-positive area was measured and related to the total area of tissue on the slide. The elastic fiber content is relative to the total tissue and served as a surrogate parameter for elastic fiber density (elastic fiber fraction). Similarly, the collagen content was related to the total tissue area and served as an indicator of fibrosis (collagen fraction). Large bronchioles and vessels were avoided for imaging and not well-inflated tissue areas were excluded from the analysis. All images for calculation were taken at 40 × magnification using a bright field microscope (Olympus, Hamburg, Germany).

### Cell culture experiments

#### Murine macrophages

Murine ascites macrophages (J774A.1, ATCC® TIB-67™) were cultured with DMEM (Dulbecco's Modified Eagle Medium, Gibco, #41966–029, Netherlands) supplemented with 10% FBS (serum-rich medium) and 1% Penicillin/Streptomycin (P/S) according to the recommendations of ATCC.

#### Human monocyte-derived macrophages and differentiation

Human blood was collected from the hospital bank (for ethical consent see below).

#### Human M1-like macrophages

Isolation of monocyte-derived macrophages was performed as described previously [62]. In brief, a buffy coat from the blood was collected from the hospital blood bank of the University Hospital Cologne (approval No 06.062) and a magnetic beads-containing antibody against CD14 cell marker was used. CD14^+^ cells were collected and kept in a serum-rich medium (RPMI supplemented with 10% FBS and 1% P/S). For differentiation in M1-like macrophages, CD14^+^ cells were treated with granulocyte–macrophage colony-stimulating factor (GM-CSF; 100 ng/ml; ImmunoTools, 11343123, Friesoythe, Germany) for 7 days; the media was changed on alternate days. At the end of 7 days, M1-like macrophages were cultured with GM-CSF in serum-reduced medium (1% FBS) and exposed to normoxia (21% O_2_) or hyperoxia (85% O_2_) for 48 h. At the end of the experiments, mRNA was isolated and gene expression was assessed by qRT-PCR.

#### M0 macrophages

Human macrophages were isolated from peripheral blood mononuclear cells (PBMCs) as previously described [63, 64]. Briefly, ficoll density gradient centrifugation was used for the isolation of PBMCs from buffy coats provided by the blood bank of the Universities of Giessen and Marburg Lung Center (AZ 58/15). Red blood cell (RBC) lysis buffer (BD Biosciences) was used to remove platelets and RBC followed by two washing steps with phosphate-buffered saline (PBS). Finally, macrophages were differentiated from monocytes by culturing cells in RPMI containing 2.5% human serum, 4 mML-glutamine, and penicillin/streptomycin in six-well tissue culture plates for 10 days. Next, we subjected for migration assay.

#### Macrophage assays and reagents

##### Migration assay

When murine or human M0-like macrophages were 80% confluent, they were trypsinized and used for the Boyden chamber migration assay. To this end, 100.000 cells were seeded per cell culture insert with serum-free DMEM medium. Subsequently, the inserts (upper chamber) were placed in a 24-well plate (lower chamber) with DMEM supplemented with 1% FBS + 1% P/S.

##### Reagents

For assessment of migration, the following treatments were used: (i) CXCL10 (10 ng/ml; R&DSystems, #466-CR050, Minneapolis, USA) was added to the well (lower chamber), (ii) CXCR3 antagonist alone (300 nm/ml; Merckmillipore, #PS372424, Darmstadt, Germany) was added to the insert; (iii) CXCR3 antagonist (300 nm/ml; Merckmillipore, #PS372424, Darmstadt, Germany) was added to the insert (upper chamber) and CXCL10 (10 ng/ml) to the well (lower chamber); (iv) DMSO served as vehicle control. Macrophages were allowed to migrate for 24 h. At the end of the experiment, H&E staining of the transwell membrane was performed to visualize; macrophages were counted per field of view, a total of eight fields of view per transwell membrane.

### In situ hybridization of human lungs

Fluorescent in situ hybridization was conducted as per manufacturer’s instructions (the Advanced Cell Diagnostics RNAscope Fluorescent Multiplex Assay) with minor adjustments as described previously [8]. First, tissue sections were treated with Protease Plus. Next, tissue was incubated with a CXCL10 probe (Advanced Cell Diagnostics; #311851) for 2 h at 40 °C on protocol day 1. Afterward, section was washed with RNAscope wash buffer, and stored overnight at RT in 5X SSC. On day 2 the protocol was continued as described in the manual until the HRP blocker step was completed. Slides were then washed with RNAscope wash buffer and blocked using 3% bovine serum albumin/5% normal goat serum/0.1% Triton for at least 1 h at RT, and incubated overnight at 4 °C with primary mouse anti-CD68 (eBioscience, #14–0688-82; 1:200, San Diego, USA). The next day slides were washed with TBST and incubated for 1 h at RT with Cy5-goat-anti-mouse-conjugated secondary antibody (Jackson Immunoresearch Laboratories, Inc., West Grove, PA, USA). Slides were then counterstained using DAPI (DE571; LifeTechnologies) and mounted using ProLong Diamond Antifade Mountant (LifeTechnologies).

### Statistical analysis

All measurements are expressed as mean ± standard error of the mean (SEM) and *p* < 0.05 denoted significant differences. To compare outcomes between two groups, we used the unpaired t-test or the Mann Whitney test. We used two-way or one-way analysis of variance followed by Sidak’s multiple comparison test if there were more than two groups. For human studies, we performed a linear regression analysis. For statistical analysis, we use the GraphPad Prism software (Software version 8.3.0).

### Supplementary Information

**Additional file 1.**

**Additional file 2.**

## Data Availability

The datasets analysed during the current study are available in the Dryad repository (https://doi.org/10.5061/dryad.rr4xgxd8m).
