# Supplementary material for: CXCL10 deficiency limits macrophage infiltration, preserves lung matrix, and enables lung growth in bronchopulmonary dysplasia
Source: Inflamm Regen. 2023 Oct 24;43:52. doi: 10.1186/s41232-023-00301-6 (PMC10594718; doi:10.1186/s41232-023-00301-6)
Supplement: Supplementary file 1 — Additional file 1. [file 41232_2023_301_MOESM1_ESM.zip › Hirani et al.,supplementary file_R1_FINAL.docx]

**CXCL10 deficiency limits macrophage infiltration, preserves lung matrix, and enables lung growth in bronchopulmonary dysplasia**

*Dharmesh V. Hirani^1,2^, *Florian Thielen^1^, Siavash Mansouri^3^, Soula Danopoulos^4^, Christina Vohlen^1,2,5^, Pinar Haznedar-Karakaya^1^, Jasmine Mohr^1^, Rebecca Wilke^1^, Jaco Selle^1^, Thomas Grosch^1^, Ivana Mizik^1^, Margarete Odenthal^6,7^, Cristina M. Alvira^8^, Celien Kuiper-Makris^1,6^, Gloria S. Pryhuber^9^, Christian Pallasch^10^, S. van Koningsbruggen-Rietschel^5^, Denise Al-Alam^4^, Werner Seeger^2,3^, Rajkumar Savai^2,3^, Jörg Dötsch^5^, Miguel A. Alejandre Alcazar^1,2,6,11^

*equally contributed

^1^Translational Experimental Pediatrics, Experimental Pulmonology, Department of Pediatric and Adolescent Medicine, University Hospital Cologne, Faculty of Medicine, University of Cologne, Cologne, Germany; ^2^Institute for Lung Health (ILH) and Cardio-Pulmonary Institute (CPI), Universities of Giessen and Marburg Lung Center (UGMLC), member of the German Center for Lung Research (DZL), Gießen, Germany; ^3^Department of Lung Development and Remodeling, Max-Planck-Institute for Heart and Lung Research, Member of the German Center for Lung Research (DZL), Bad Nauheim, Germany; ^4^Lundquist Institute for Biomedical Innovation at Harbor-UCLA Medical Center, Torrance, CA, USA; ^5^Department of Pediatric and Adolescent Medicine, University Hospital Cologne, Faculty of Medicine, and University of Cologne, Cologne, Germany; ^6^Center for Molecular Medicine Cologne (CMMC), University Hospital Cologne, Faculty of Medicine, and University of Cologne, Cologne, Germany; ^7^Institute for Pathology, University Hospital Cologne, Faculty of Medicine, and University of Cologne, Cologne, Germany; ^8^Department of Pediatrics, Stanford University School of Medicine, Stanford, CA, USA; ^9^Division of Neonatology, Department of Pediatrics, University of Rochester Medical Center, Rochester, NY, USA; ^10^Department I of Internal Medicine, Center for Integrated Oncology (CIO) Köln-Bonn, University of Cologne, Cologne, Germany; ^11^Cologne Excellence Cluster on Stress Responses in Aging-associated Diseases (CECAD), University Hospital of Cologne, University of Cologne, Cologne, Germany.

**Supplementary figures**

**Supplementary Figure 1:** A-G: Measurement of gene expression of collagen 1 alpha 1 (*Col1a1*; A), *Col3a1* (B), *Col4a4* (C), tumor necrosis factor alpha (*Tnfa*; D), interleukin 1 beta (*Il1b*; E), arginase 1 (*Arg1*; F), and *Il4* (G) in total lung homogenates of wildtype (WT) and *Cxcl10* knockout mice (*Cxcl10*^-/-^) at postnatal 14 (P14) after exposure to normoxia (NOX, 21% O_2_) or hyperoxia (HYX, 85% O_2_). Mean±SEM; n = 6-9/group. Two-way ANOVA: *p<0.05, **p<0.01, ****p<0.0001.

**Supplementary Figure 2:** A-C: Measurement of gene expression of collagen 1 alpha 1 (*Col1a1*; A), Col3a1 (B), and Col4a4 (C) in total lung homogenates of wildtype (WT) and *Cxcl10* knockout mice (*Cxcl10*^-/-^) at postnatal 28 (P28) after exposure to normoxia (NOX, 21% O_2_) or hyperoxia (HYX, 85% O_2_). Mean±SEM; n = 8-10/group. Two-way ANOVA: *p<0.05.

**Supplementary Figure 3:** Measurement of gene expression of *Cxcl10* in lungs of wildtype (WT) and Cxcl10 knockout mice (*Cxcl10*^-/-^) at postnatal 28 (P28) after exposure to normoxia (NOX, 21% O_2_) or hyperoxia (HYX, 85% O_2_) from birth until P14. Mean±SEM; n = 10-11/group. Mann-Whitney test: ^##^p<0.01.

**Supplementary Figure 4:** A-D: Measurement of gene expression of tumor necrosis factor alpha (*Tnfa*; D), interleukin 1 beta (*Il1b*; E), arginase 1 (*Arg1*; F), and *Il4* (G) in total lung homogenates of wildtype (WT) and *Cxcl10* knockout mice (*Cxcl10*^-/-^) at postnatal 14 (P14) after exposure to normoxia (NOX, 21% O_2_) or hyperoxia (HYX, 85% O_2_). Mean±SEM; n = 8-10/group. Two-way ANOVA: *p<0.05.

**Supplementary Figure 5:** A, B: Representative images of migrated J774A.1 murine macrophages treated with vehicle (DMSO) or CXCR3 antagonist (300 nm/ml) for 24 h. Quantification of migrated macrophages per field of view (20X). C, D: Representative images of migrated human M0-like macrophages treated with either only CXCL10 (10 ng/ml), CXCL10 plus CXCR3 antagonist (300 nm/ml) or CXCR3 antagonist alone (300nm/ml) for 24h; controls were treated with DMSO. Quantification of migrated human M0-like macrophages per field of view (20X). Mean±SEM; n = 3-4/group; Mann-Whitney test: not significant; One-way ANOVA: ***p<0.001; ****p<0.0001.

Supplementary Table 1.

| **Up regulated genes** | | |  | **Down regulated genes** | | |
| --- | --- | --- | --- | --- | --- | --- |
| **Gene_symbol** | **Foldchange** | **PValue** |  | **Gene_symbol** | **Foldchange** | **PValue** |
| Mmp10 | 1105,58807 | 1,45E-46 |  | Prokr2 | -6,209459896 | 1,61E-08 |
| Ano3 | 172,926144 | 2,05E-85 |  | Kit | -6,307210689 | 1,37E-67 |
| Fosl1 | 69,2224688 | 6,08E-21 |  | Vstm2b | -6,38631584 | 8,95E-08 |
| Nmrk2 | 43,4437131 | 1,30E-21 |  | Cpa1 | -6,480830155 | 2,81E-14 |
| Cxcl5 | 42,2340011 | 2,66E-30 |  | 9330159F19Rik | -6,598302807 | 1,64E-18 |
| Ccl20 | 38,3898714 | 1,10E-23 |  | Vtn | -6,672984647 | 2,29E-45 |
| Inhba | 33,5670541 | 1,14E-45 |  | Sap25 | -6,835771114 | 0,04928909 |
| Chodl | 28,880827 | 1,60E-34 |  | Cilp | -6,845004914 | 5,56E-10 |
| Acta1 | 26,2245429 | 7,90E-12 |  | Lrrtm3 | -6,870272819 | 9,55E-17 |
| Ccl2 | 21,4176508 | 6,05E-24 |  | Ntrk2 | -6,943774674 | 3,62E-29 |
| Eda2r | 20,3295678 | 1,07E-69 |  | Ear1 | -7,065641195 | 4,32E-20 |
| Cxcl10 | 19,8924373 | 6,56E-13 |  | Hpca | -7,177228712 | 1,83E-12 |
| Saa3 | 16,9817343 | 2,70E-15 |  | Dlk1 | -7,492284133 | 3,43E-05 |
| Gdf15 | 13,7074938 | 7,14E-47 |  | Vsnl1 | -7,573711341 | 8,15E-135 |
| Gdf6 | 12,9630304 | 1,45E-21 |  | Cidec | -7,620998607 | 5,22E-11 |
| Cdkn1a | 12,6361171 | 7,90E-122 |  | Abca17 | -7,717476426 | 3,73E-17 |
| Nppa | 10,9748562 | 1,73E-05 |  | Adcy8 | -7,814788582 | 5,08E-43 |
| Gm11974 | 10,8619271 | 1,03E-39 |  | Slc26a10 | -8,218989358 | 2,40E-19 |
| Arl14 | 10,3674509 | 9,81E-19 |  | Havcr1 | -8,892621715 | 3,03E-23 |
| Ptprn | 10,1311708 | 2,52E-43 |  | Glp1r | -8,939691859 | 5,16E-28 |
| Serpina3n | 9,6334911 | 2,71E-31 |  | Zfp663 | -9,092907981 | 8,07E-19 |
| Sall1 | 9,61727902 | 2,97E-29 |  | D330041H03Rik | -9,303739123 | 0,00024653 |
| Psrc1 | 8,53042022 | 1,34E-79 |  | Glb1l3 | -11,23464401 | 7,40E-17 |
| Pigr | 8,12047019 | 6,86E-12 |  | Fam163a | -12,17687236 | 2,16E-12 |
| Ccl7 | 8,09679071 | 3,55E-12 |  | Crabp1 | -29,92608702 | 6,48E-24 |

Supplementary Table 2.

| **Gene** | **Species**  **m = mouse**  **h = human** | **Primer** |
| --- | --- | --- |
| *18s rRNA* | m | for AGATCCCAGACTGGTTCCTG  rev TTGTTGTCTAGACCGTTGGC  teq CAGAACCTGGCTGTACTTCCCATCC |
| *Arg1* | m | for ACCCTGACCTATGTGTCATTTGG  rev TGGTACATCTGGGAACTTTCCTTT  taq ATGCTCACACTGACATCAACACTCCCCTG |
| *Ccl2* | m | for GGCTCAGCCAGATGCAGTTAAC rev CTTGGTGACAAAAACTACAGCTTCTT teq CCCCACTCACCTGCTGCTACTCATTCA |
| **C1qb* | m | for AAGATCCAGAAACACAAGTCCCT  rev CCTCCTCACCATCAAATGTTGG |
| **Col1a1* | m | for GCAGTGCTGTTGCGATCTTG  rev CAGAGGGACAGAGCACAGCTT |
| **Col3a1* | m | for GGTGGTTTTCAGTTCAGCTATGG  rev TTTTTGCAGTGGTATGTAATGTTCTG |
| *Col4a4* | m | for GAAGGCGCACAATCAAGATCT  rev CACACTTGGTGGATGTTGCAGTA  taq CTTCCTGTGTTTAGCACTCTGCCCTTTGC |
| *Cxcl10* | m | for CATCCCTGCGAGCCTATCC  rev CCCTTTTAGACCTTTTTTGGCTAA  taq CCCACGTGTTGAGATCATTGCCACG |
| **Cxcr3* | m | for GCTGCTGTCCAGTGGGTTTT  rev GGAAGGCCCCTGCATAGAAG |
| **Ilb* | m | for TGACAGTGATGAGAATGACCTGTTC  rev GGACAGCCCAGGTCAAAGG |
| **Il4* | m | for GGAGATGGATGTGCCAAACG  rev GCACCTTGGAAGCCCTACAG |
| *Il6* | m | for ACAAGTCGGAGGCTTAATTACACAT rev AATCAGAATTGCCATTGCACAA teq TCTTTTCTCATTTCCACGATTTCCCAGAGAA |
| **Lgals3* | m | for TGCTGGTTCCAGGGACTCAA  rev CCACCGGCCTCTGTAGAAGA |
| *Nos2* | m | for CAGTGGAGAGATTTTGCATGACA  rev GGGTCCTCTGGTCAAACTCTTG  taq CACCACAAGGCCACATCGGATTTCAC |
| *Tnfa* | m | for AGGGATGAGAAGTTCCCAAATG  rev GCTTGTCACTCGAATTTTGAGAAG |
| *18s rRNA | h | for CGGCTACCACATCCAAGGAA  rev GCTGGAATTACCGCGGCT |
| *CCL2 | h | for CAGCCACCTTCATTCCCCAA  rev GGACACTTGCTGCTGGTGAT |
| *CXCL10 | h | for TGAAAAAGAAGGGTGAGAAGAGATG  rev TTTAGACCTTTCCTTGCTAACTGCTT |
| *IL1b | h | for CTAAACAGATGAAGTGCTCC rev GGTCATTCTCCTGGAAGG |
| *NOS2 | h | for GCAGGTCGAGGACTATTTCTTTCA  rev CGTAAGGAAATACAGCACCAAAGAT |

Supplementary table 2. List of primers used for real-time RT-PCR; *SYBR-Green primer.
