# Supplementary figures and images for: CXCL10 deficiency limits macrophage infiltration, preserves lung matrix, and enables lung growth in bronchopulmonary dysplasia
Source: Inflamm Regen. 2023 Oct 24;43:52. doi: 10.1186/s41232-023-00301-6 (PMC10594718; doi:10.1186/s41232-023-00301-6)

supplementary figure 1

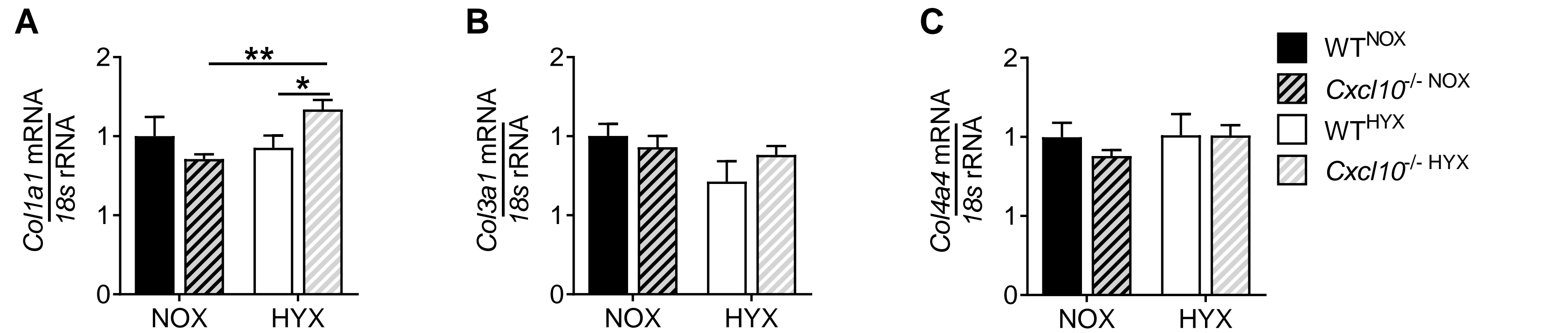

M1-like macrophage markers

M2-like macrophage markers

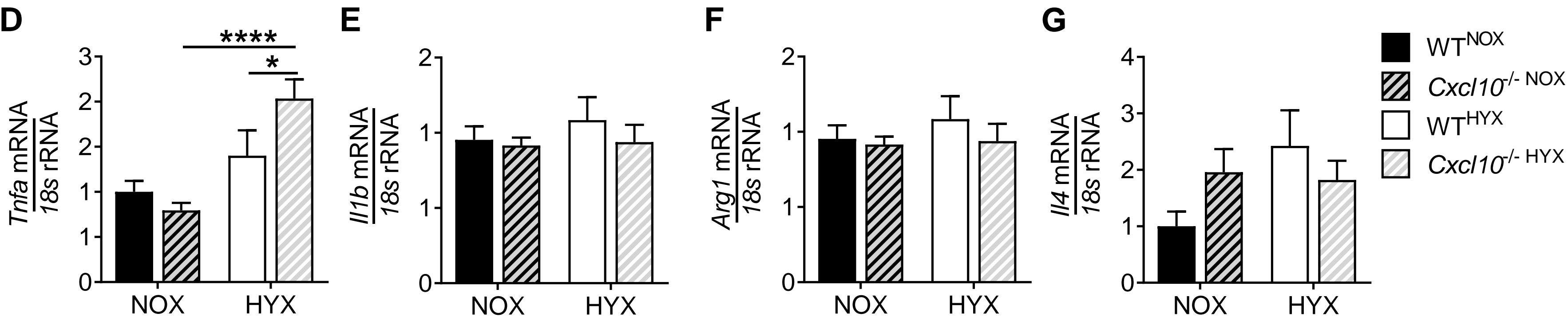

Supplement: Supplementary file 1 — Additional file 1. [file 41232_2023_301_MOESM1_ESM.zip › Supplementary Figure 1.pdf]

## Supplementary figure 2

**A**

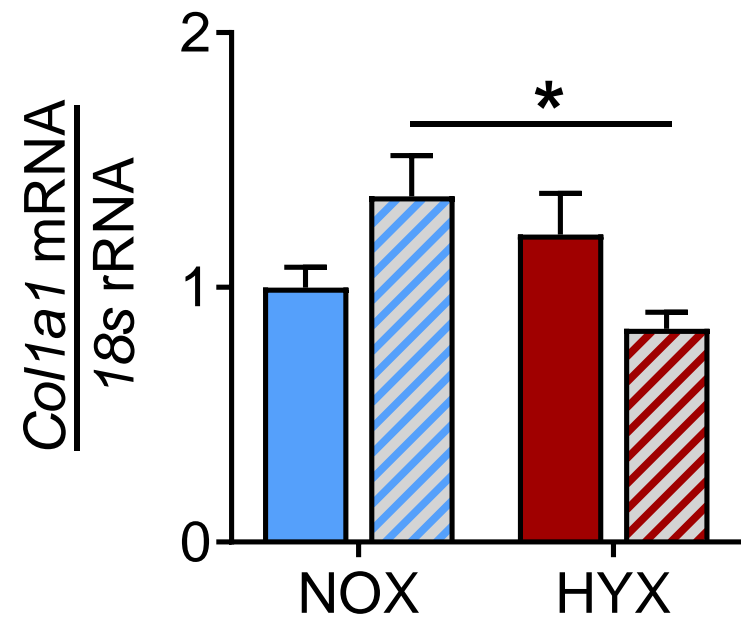

**B**

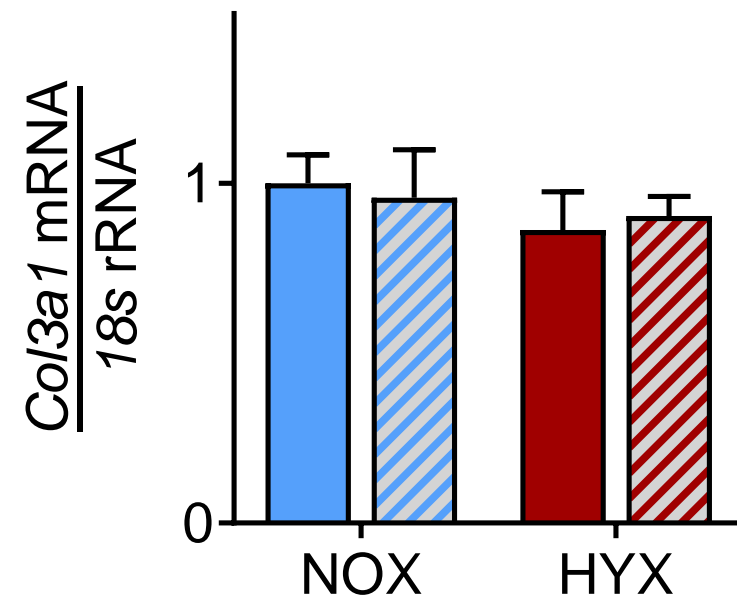

**C**

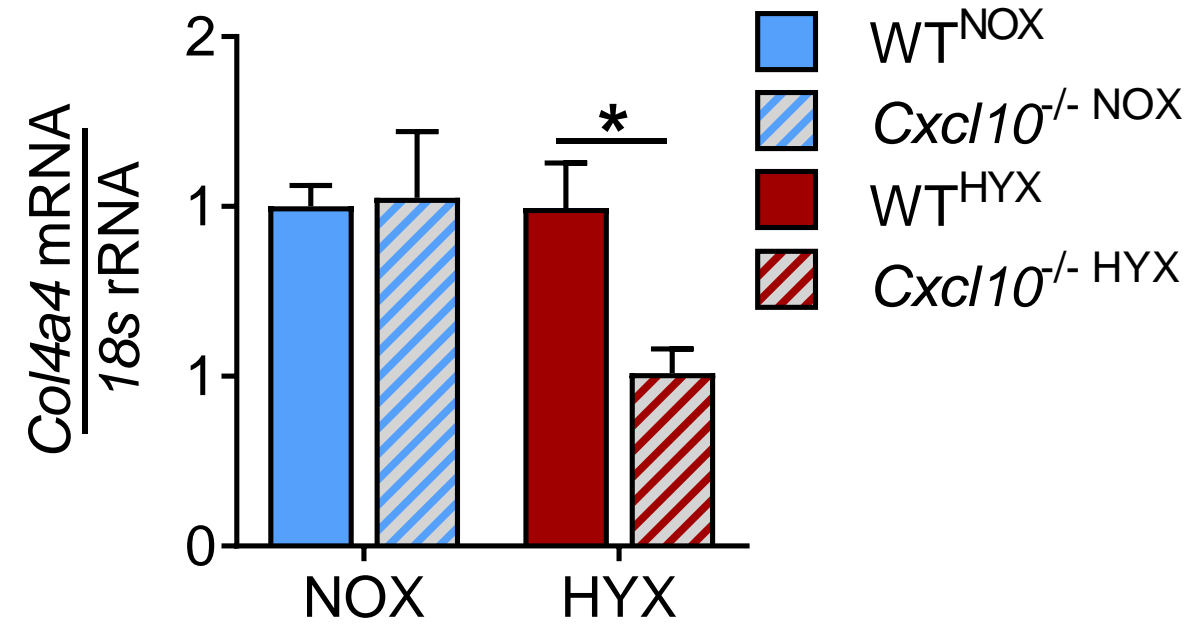

Supplement: Supplementary file 1 — Additional file 1. [file 41232_2023_301_MOESM1_ESM.zip › Supplementary Figure 2.pdf]

# Supplementary figure 3

**A**

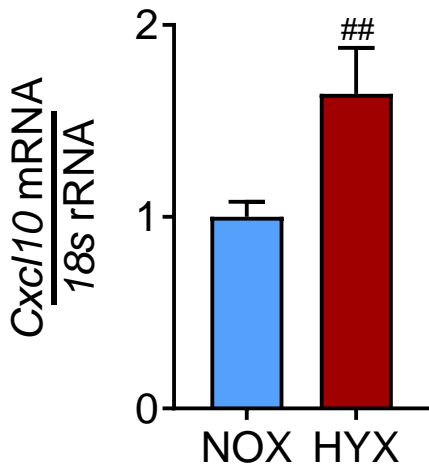

Supplement: Supplementary file 1 — Additional file 1. [file 41232_2023_301_MOESM1_ESM.zip › Supplementary Figure 3.pdf]

# Supplementary figure 4

## M1-like macrophage markers

## M2-like macrophage markers

**A**

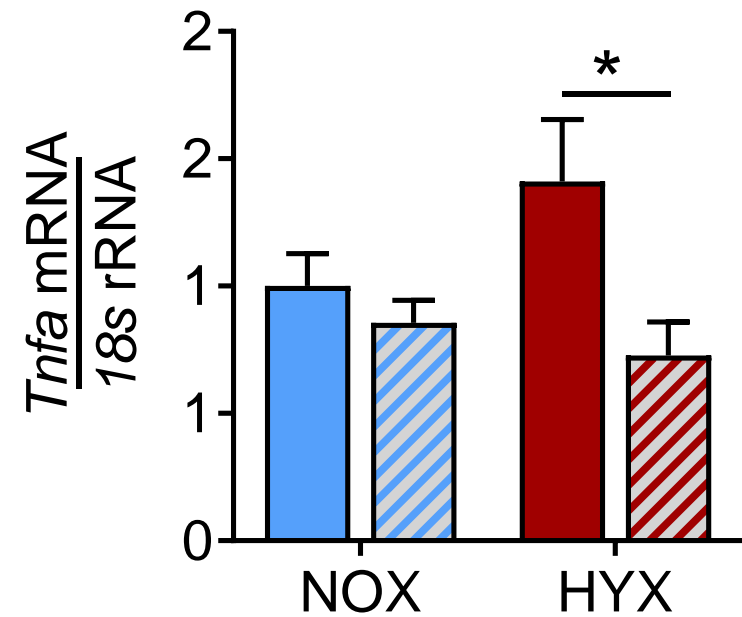

**B**

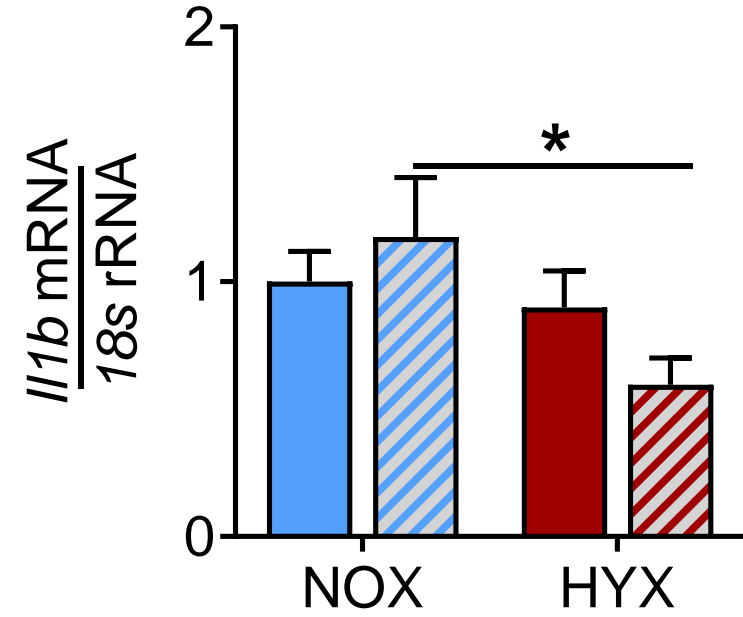

**C**

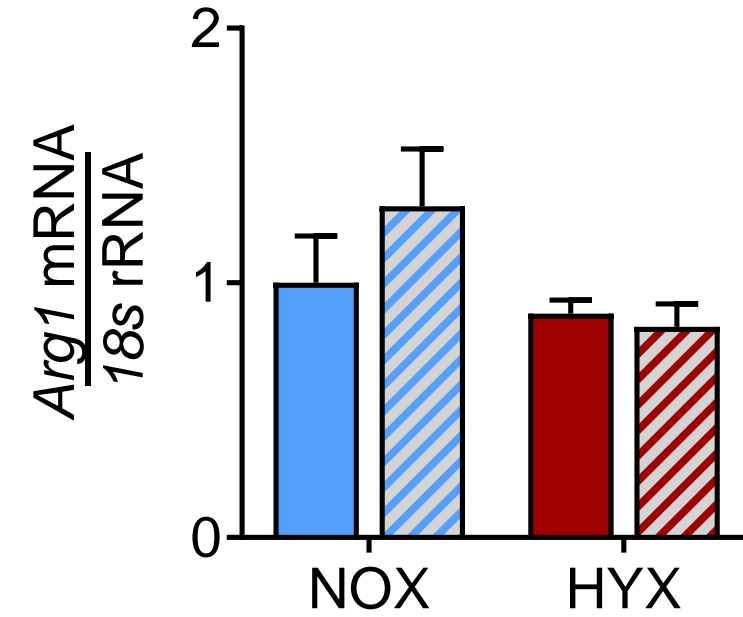

**D**

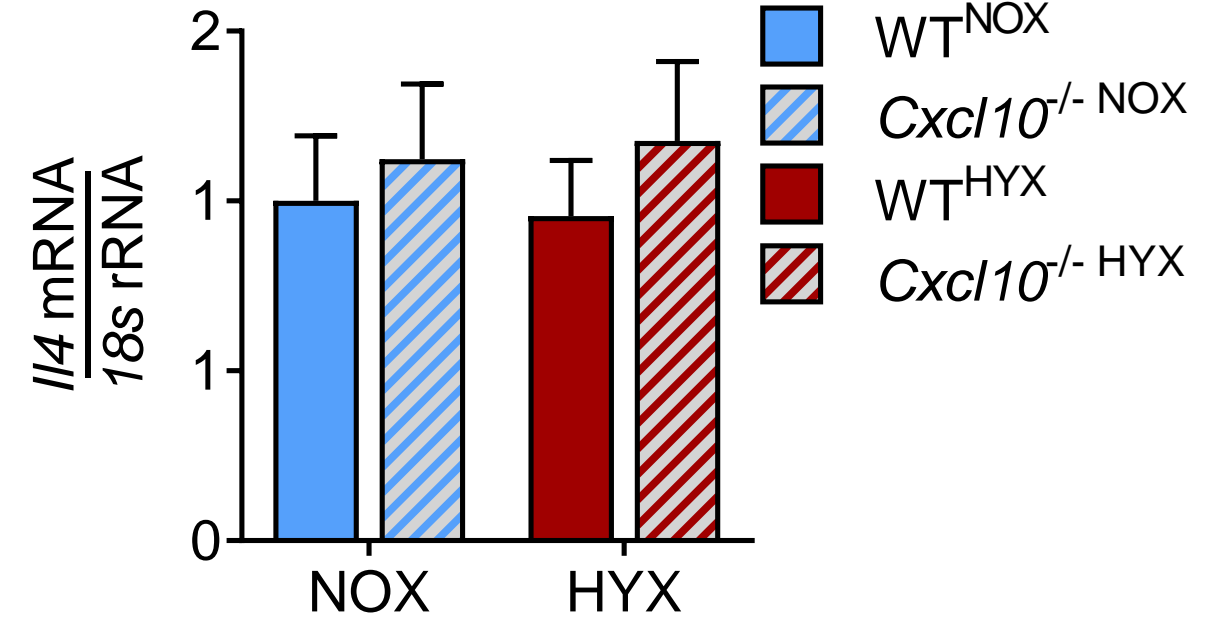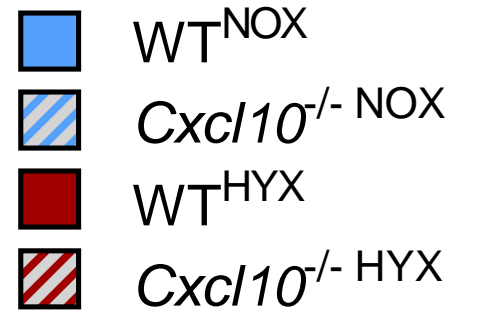

Supplement: Supplementary file 1 — Additional file 1. [file 41232_2023_301_MOESM1_ESM.zip › Supplementary Figure 4.pdf]

Supplementary figure 5

A

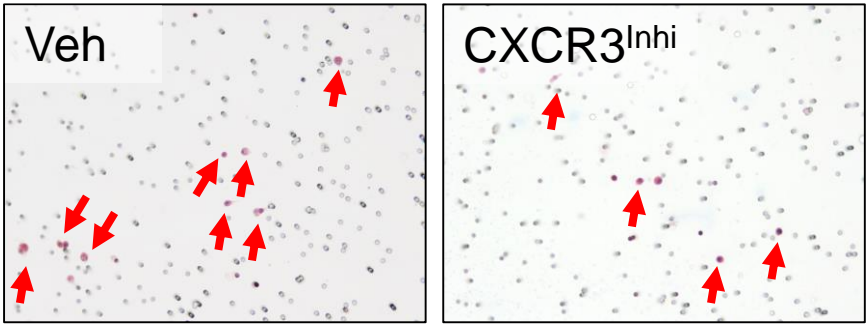

B

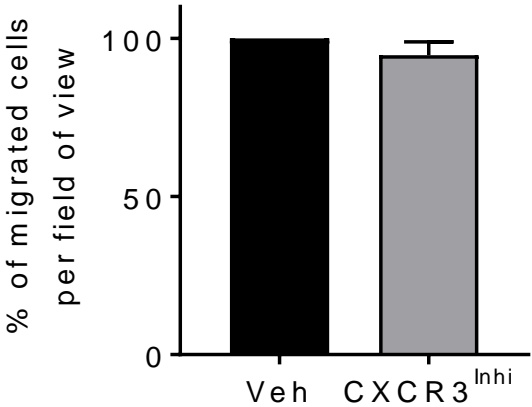

C

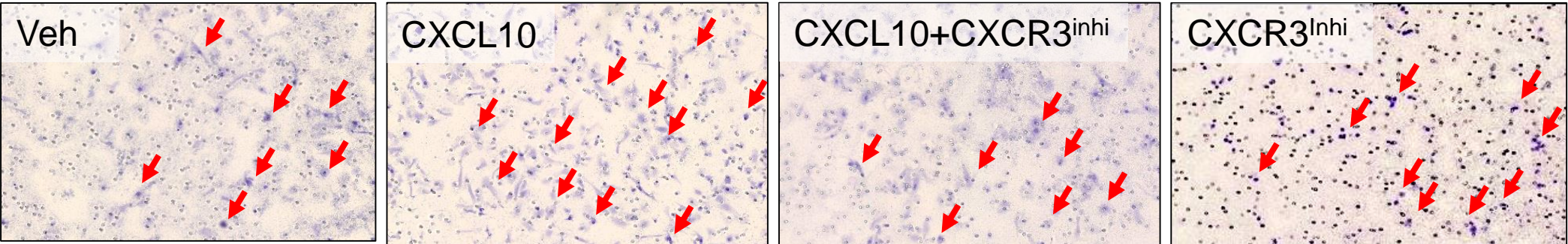

D

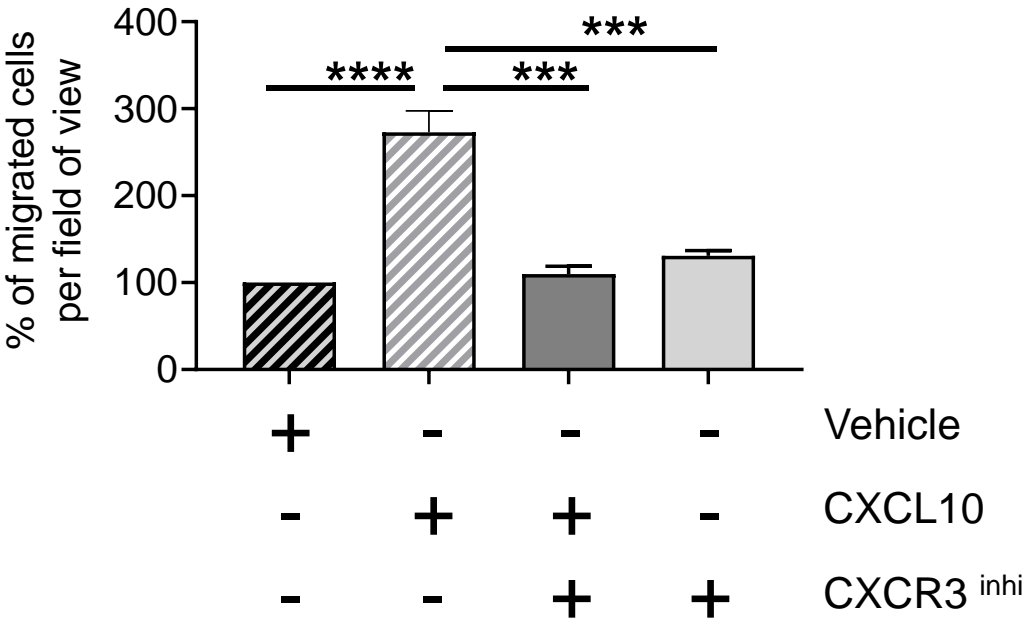

Supplement: Supplementary file 1 — Additional file 1. [file 41232_2023_301_MOESM1_ESM.zip › Supplementary Figure 5.pdf]
